# Strong Families Study: protocol for a co-designed birth cohort study with Aboriginal and Torres Strait Islander families in Queensland, Australia

**DOI:** 10.1136/bmjopen-2025-113766

**Published:** 2026-05-28

**Authors:** Salma Mohamed Ahmed, Emily S Dorey, Davina Smith, Loretta Weatherall, Rhiannon Friday, Luciana F Massi, Rebecca Rooney, Diana Hermith-Ramirez, Emma Kendall, Kai Wheeler, Anne-Marie Eades, Maree Toombs, Roslyn N Boyd, Rhonda Marriott, Sandra J Eades, Katherine Benfer, Natasha Reid, Koa Whittingham, Robert S Ware, Elizabeth Martin, Paul D Robinson, Vicki Clifton, Leonie Kaye Callaway, Sailesh Kumar, Kym M Rae, The Strong Families Indigenous Steering Committee, Sandra Eades

**Affiliations:** 1 Indigenous Health Research Group, Mater Research Institute, The University of Queensland, South Brisbane, Queensland, Australia; 2 Queensland Cerebral Palsy and Rehabilitation Research Centre, Child Health Research Centre, Faculty of Health, Medicine and Behavioural Sciences, The University of Queensland, Brisbane, Queensland, Australia; 3 Griffith University, Brisbane, Queensland, Australia; 4 School of Human Movement and Nutrition Sciences, Centre for Research on Exercise, Physical Activity and Health, The University of Queensland, Brisbane, Queensland, Australia; 5 Curtin School of Nursing, Curtin University, Perth, Perth, Australia; 6 Faculty of Medicine and Health, University of New South Wales, Sydney, New South Wales, Australia; 7 Faculty of Health, Medicine and Behavioural Sciences, The University of Queensland, Brisbane, Queensland, Australia; 8 Ngangk Yira Centre for Change, Murdoch University, Murdoch, Western Australia, Australia; 9 School of Population and Global Health, Faculty of Medicine, Dentistry and Health Sciences, The University of Melbourne, Melbourne, Victoria, Australia; 10 Wesley Research Institute, Brisbane, Queensland, Australia; 11 Duke-NUS Medical School, Singapore; 12 Children’s Health and Environment Program, Child Health Research Centre, The University of Queensland, Brisbane, Queensland, Australia; 13 Pregnancy and Development Group, Mater Research Institute, The University of Queensland, South Brisbane, Queensland, Australia; 14 Women’s and Newborn Services, Royal Brisbane and Women’s Hospital, Metro North Health, Herston, Queensland, Australia; 15 Maternal & Fetal Medicine, Mater Research Institute, The University of Queensland, South Brisbane, Queensland, Australia

**Keywords:** Australian Aboriginal and Torres Strait Islander Peoples, Longitudinal studies, Community-Based Participatory Research, Child, Family, Pregnancy

## Abstract

**Abstract:**

**Introduction:**

Australian studies investigating parental factors often lack meaningful inclusion of Aboriginal and Torres Strait Islander families, limiting our understanding of current influences on positive developmental trajectories within communities. There is growing recognition of the need for culturally safe and responsive longitudinal research that is co-designed and co-led by the community for the community. An Indigenous-led birth cohort study of Aboriginal and Torres Strait Islander families in Queensland, Australia, has therefore been developed to better understand health across generations.

**Methods and analysis:**

The Strong Families Study is a co-designed prospective longitudinal birth cohort study that will follow 400 Indigenous families in Queensland from pregnancy until the child reaches 5 years of age. Eligible participants include pregnant individuals (<28 weeks’ gestation) whose children may identify as Aboriginal and/or Torres Strait Islander, along with their partners (if applicable). Data will be collected at multiple timepoints: during gestation, at delivery, postpartum, every 6 months until the child is 36 months (corrected age, CA) and annually until age 5 years. These will be collected by Aboriginal health workers using validated and culturally appropriate tools across different health themes. The study will incorporate health literacy throughout, as well as referrals to two nested family support programmes to support families and assist with the developmental outcomes of their children when and if required. Effects across health themes will be analysed with a focus on strengths, positive trajectories and holistic well-being of Indigenous families, moving beyond deficit-based narratives.

**Ethics and dissemination:**

This study was approved by the Mater Misericordiae Ltd Human Research Ethics Committee (HREC/MML/105191) and ratified by the University of Queensland Human Research Ethics Committee (2025/HE001924). Endorsement letters were secured from partner services at each study site. Findings will be shared with partnering hospitals and funding bodies at conferences and through reports and peer-reviewed publications.

**STRENGTHS AND LIMITATIONS OF THIS STUDY**

- This co-designed birth cohort study integrates Indigenous knowledge systems into its design and uses strength-based approaches that emphasise positive trajectories within Indigenous families.
- The inclusion of both partners in the birth cohort study, as well as the collection of biological samples from all participants, offers novel insights into family health determinants.
- Multiple in-person visits, from pregnancy through birth and childhood, may be burdensome for participants and increase the risk of attrition.
- Restricting enrolment to individuals <28 weeks’ gestation may limit generalisability of findings.

## Introduction

 Globally, Indigenous peoples constitute over 6% of the population, with an estimated 476.6 million individuals across all regions.^1^ For millennia, these populations have recognised the profound impact of past generations on shaping the life experiences of the present. This perspective aligns with the Developmental Origins of Health and Disease (DOHaD) framework, which emphasises the role of early-life environmental factors in influencing children’s development, risk factors for disease and their long-term health throughout their lifespan.^2^ The understanding of DOHaD is particularly evident in Australia, where Aboriginal and Torres Strait Islander (hereafter respectfully ‘Indigenous’) people have the oldest continuing culture in the world, with Aboriginal knowledge systems having survived for over 60 000 years.^3^ As such, both the strength of Indigenous peoples and their cultural traditions have enabled them to persist and rise through the lasting impacts of colonisation. The maintenance of strong and cohesive identity and cultural practices including the centrality of country, community and family, underpinned by self-determination, has been identified as positively impacting the health and well-being of Indigenous peoples.^4^ These cultural foundations promote resilience and cultural continuity, contributing to positive outcomes for Indigenous families and the broader well-being of communities. Among Indigenous peoples worldwide, the concept of health is characterised by a holistic framework that integrates distinct yet interconnected dimensions.^5^ In the Australian context, Indigenous people define health as encompassing the physical, spiritual, cultural and social well-being of both individuals and their communities.^6^

The period of time from conception to age 5, being the first 2000 days of a child’s life, represents a critical developmental window which is a key focus of the DOHaD hypothesis. During this time, children undergo significant cognitive, social, emotional and physical growth, and their early experiences have long-lasting impacts on future health and well-being.^7^ The first 2000 days are increasingly recognised in academic, policy and mainstream health literature as a pivotal period that influences lifelong outcomes.^8^,^11^ Such research consistently demonstrates that early-life interventions and supports during this period are central to promoting positive parent-child relationships and developmental outcomes, particularly for populations that continue to experience the effects of colonisation. Given the influence of early experiences, exploring strategies and interventions to support children during the first 2000 days is critical for ensuring better health and developmental trajectories for future generations.

Among the limited research available, several key longitudinal studies have followed the health and development of Indigenous mothers and children in Australia. The Aboriginal Birth Cohort study (n=686 Aboriginal maternal-child pairs) in the Northern Territory is the largest and longest running of its kind.^12^ Other notable studies include the Gudaga Study^13^ in urban Sydney, which followed mother-infant pairs (n=155) through the first year; the Gomeroi Gaaynngal Study^14^ in rural and regional New South Wales, tracking participants from pregnancy through childhood (n=110 mother-infant dyads); and the PANDORA Study,^15^ which examines outcomes for Indigenous women (n=542), particularly those with diabetes during pregnancy in the Northern Territory. Despite these efforts, there are currently no birth cohort studies involving Australian Indigenous families that have research priorities and methodologies co-designed in partnership with Indigenous community members. Furthermore, to our knowledge, no existing birth cohort studies have actively included both Aboriginal and Torres Strait Islander fathers or second parents in their design and data collection.

Co-design underscores the importance of collaborating with key stakeholders through capability building, sharing decision-making, and the development of equitable, sustained partnerships.^16 17^ Co-design values consumer-driven research and engagement throughout all stages of the research process, using Aboriginal Participatory Action Research to ensure Indigenous voices are central and the study remains relevant and aligns with community priorities and needs.^17^,^20^ The integration of an Indigenous lens within these processes facilitates the recognition and integration of Indigenous knowledge systems and the opportunity for cultural considerations that have historically been overlooked in previous policies and mainstream research.^21^ Furthermore, such approaches can counter the pervasive deficit discourse research relating to Indigenous health and well-being, which are often negative and disempowering.^22 23^ By using Indigenous voices and lived experiences, co-design highlights the strengths and resilience of Indigenous peoples, framing these as essential pathways to self-determination and sovereignty rights.^24 25^ This co-design approach ensures the development and application of culturally sensitive methodologies that are more likely to be valued by the community they aim to serve.^18^ Overall, a co-design process underpins ethical reciprocal research, emphasising empowerment and equity to ensure meaningful, sustainable and beneficial outcomes for the community.

This paper describes the research protocol and methodology for The Strong Families Study, a co-designed, Indigenous-led, prospective longitudinal study of Indigenous families in Queensland, Australia. This birth cohort study aims to examine the perinatal strengths and risk factors among reproductive-aged Indigenous parents. Specifically, it seeks to explore associations between these parental strengths and potential risk factors and (1) maternal pregnancy outcomes, (2) neonatal and birth outcomes and (3) early childhood developmental outcomes, with the overarching goal of informing evidence-based policy and practice change. Families will be followed at multiple time points, throughout pregnancy and early childhood.

This study will be the first of its kind in Queensland, focusing on the needs of Aboriginal and Torres Strait Islander families, inclusive of both partners, particularly in the first 2000 days of a child’s life. This will be significant as Queensland has the second highest population of Indigenous people in Australia.^26^ Additionally, the study will incorporate two nested family support programmes that can support families going forward. The first programme, PACT Online,^27^ offers parents the opportunity to self-refer at any time to an online support programme grounded in acceptance and commitment therapy (ACT). The second, the Learning through Everyday Activities with Parents Study (LEAP), will establish a referral pathway for infants identified as having a higher chance of neurodevelopmental disabilities and their families.^28 29^

## Methods and analysis

### Study design and setting

The Strong Families Study (SFS) will be a longitudinal birth cohort study, with nested family support programmes designed to follow 400 Indigenous families in Queensland, Australia, from pregnancy (commencing in mid-2026) through to the child’s fifth year of life. Eligible participants will be assessed at multiple time points from pregnancy through early childhood. Comprehensive measurements and data collection, including biological samples, will be undertaken throughout the study. The measures will encompass a range of health-related themes, gathered from all participating family members.

**Figure 1:**
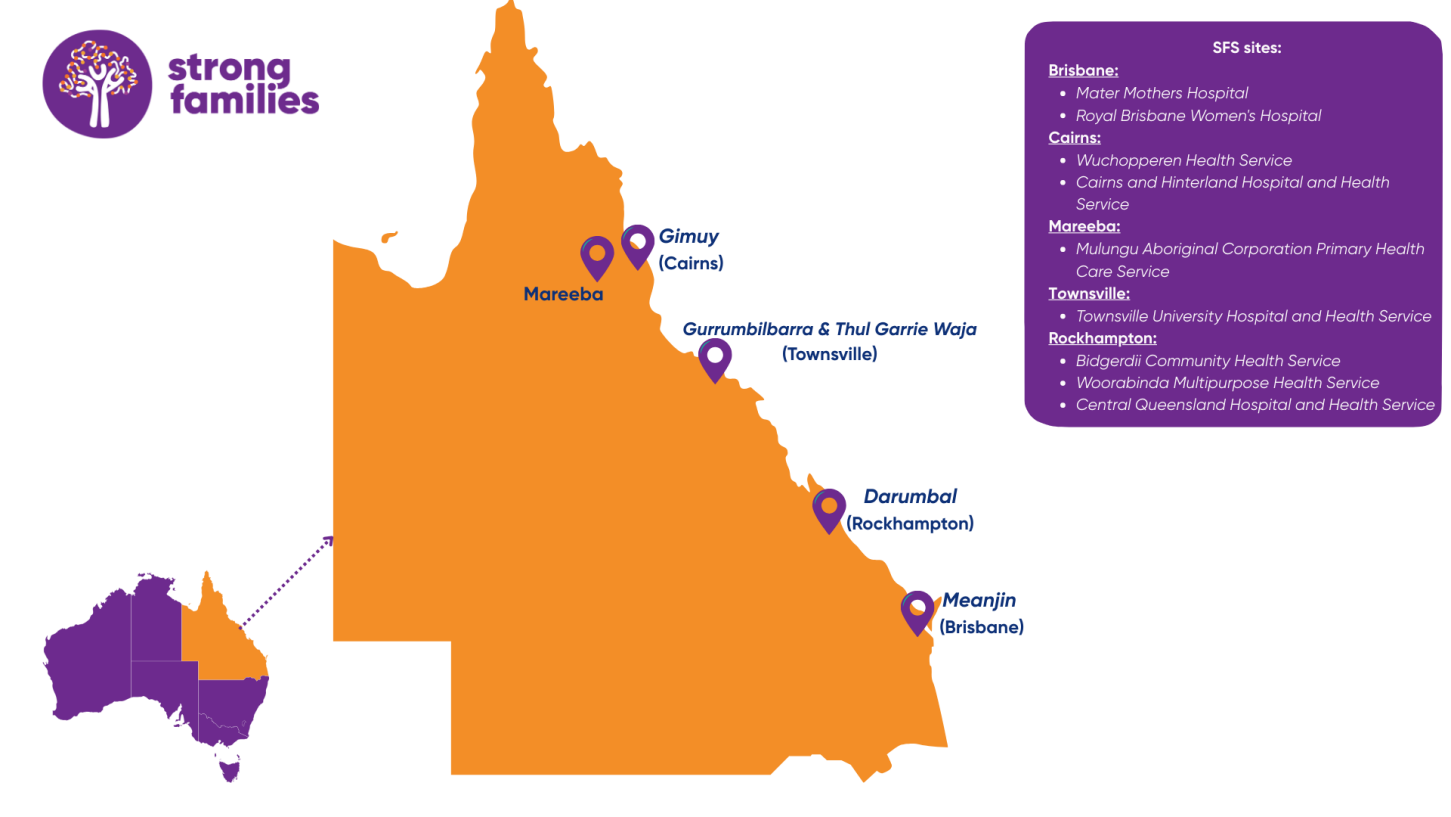
Proposed Strong Families Study sites across Queensland, Australia, with Brisbane as the coordinating centre.

The study will be conducted as a teletrial model across multiple sites (figure 1), using telehealth technology to enhance equitable access for research participants living in regional, rural or remote areas. The coordinating centre will be based at the Mater Research Institute-The University of Queensland in Brisbane. The study sites include Brisbane (metropolitan), Townsville (regional), Rockhampton (regional) and the Far North Queensland region including Cairns and Mareeba (regionals). In Brisbane, the partnering sites will be the Mater Mothers’ Hospital and Royal Brisbane Women’s Hospital, both quaternary maternity referral hospitals in Queensland. In Townsville, the study has partnered with the Aboriginal Community-Controlled Health Organisation (ACCHO), Townsville Aboriginal and Islander Health Service (TAIHS) and the Townsville University Hospital and Health Service for the protocol design. In Rockhampton, partnerships have been formed with Bidgerdii Community Health Service, Woorabinda Multipurpose Health Service and Central Queensland Hospital and Health Service. In Far North Queensland, the study has partnered with Wuchopperen Health Service, Mulungu Aboriginal Corporation Primary Health Care Service and Cairns and Hinterland Hospital and Health Service. It is likely that some smaller communities from within these regions may also take part once the study commences.

### Patient and public involvement

This study was developed through extensive community consultation and co-designed by the Strong Families Indigenous Steering Committee (ISC). The committee identified major research themes relevant to their communities, which were informed by findings from a consultation-based study called The Indigenous Health Priorities Study (I-Priorities study).^30^,^34^ The formation of the ISC itself was a direct outcome of the I-Priorities study, wherein communities identified their research needs and priorities, and the research team built rapport and trust with Indigenous community-controlled health services. This study was facilitated with support from the Queensland Aboriginal and Islander Health Council (QAIHC).^30^ With this foundation in place, the ISC was formed in late 2023 and currently has 28 representatives from across Queensland, most of whom are Aboriginal and/or Torres Strait Islander. Members include Aboriginal community representatives and study champions from over 16 health services in Queensland, reflecting diverse cultural and professional knowledge, backgrounds and expertise. In-person co-design workshops, as well as quarterly online meetings, were held with the ISC from 2023 to 2026 to develop the SFS protocol, study design, data sovereignty and other key study elements.

Throughout these in-person workshops, the ISC strongly advocated for the integration of strength-based attributes across the study. For example, Indigenous data sovereignty, the use of Aboriginal health workers to deliver surveys and the incorporation of culturally responsive, strengths-focused tools and questionnaires that reflect resilience and cultural identity. It was emphasised that data analysis and interpretation of outcomes from the study should move beyond deficit-based narratives and instead highlight positive trajectories, strengths and holistic well-being of Indigenous families. SFS builds on the Indigenous Queensland Family Cohort (I-QFC), a feasibility study conducted in Brisbane as part of the broader longitudinal Queensland Family Cohort (QFC) study.^35^ I-QFC followed Indigenous families during pregnancy and the early postpartum period. These study findings were presented to the ISC and were used to guide and inform the design and data collection frameworks of SFS.

### Study governance

The SFS is jointly overseen by the Chief Executive Committee (CEC) and the ISC, ensuring Indigenous leadership and community representation. Senior Indigenous academics participate across both committees to ensure clear communication throughout the study. The ISC’s responsibilities encompass deliberation and decision-making to ensure Indigenous community input is integrated across all phases of the research process, including cultural oversight over the study. The CEC, comprised of research academics and representatives from partner organisations, is responsible for ensuring adherence to the project timeline and budget, implementation of protocols as well as interpretation and translation of findings. Both committees will convene at least quarterly and will be governed by Terms of Reference. The Terms of Reference will be reviewed annually by the ISC. Committee membership will be extended by invitation from current ISC members and the CEC, should any existing members need to step down. This will ensure that the ISC will remain active in a governance role over time. Any modifications to the SFS protocol and sub-study applications, including access to linked data, will be reviewed by the ISC before being submitted to the CEC and the appropriate ethics committee for approval.

### Study procedure

#### Study eligibility

Pregnant individuals who are less than 28 weeks of gestation and whose children may identify as Aboriginal and/or Torres Strait Islander, along with their partner (if applicable), will be eligible to participate in the study. To ensure the cohort is representative of the broader Queensland population, adoptive families, same-sex couples, single mothers or widowed mothers will also be invited to participate, including individuals with underlying health conditions. In many regions, English may not be the first language for Indigenous communities; however, due to the large number of Indigenous languages across Queensland, participation will be limited to individuals who can understand English. Exclusion criteria include participants under 16 years of age and those unable to provide informed consent.

#### Recruitment

Initial recruitment will begin in Brisbane in mid-2026, followed by rollout to other sites shortly thereafter. Recruitment will be conducted through antenatal clinics at the listed study sites, employing a range of methods including flyers/posters, newsletters, social media and websites of partner organisations. Where feasible, experienced Indigenous health team members, such as midwives, Indigenous research assistants and Aboriginal health workers, will identify and approach potential pregnant individuals and their partners (if applicable).

Recruitment at hospitals will involve the screening of medical records to assess potential participants’ eligibility based on the study criteria, particularly that the mother is carrying an Indigenous infant in her pregnancy. This process will also ensure care is taken not to contact individuals who may have recently experienced an unexpected or adverse pregnancy complication, eg, miscarriage, stillbirth, termination or fertility issues, as this could trigger discomfort or emotional distress. Potential participants will be contacted either face-to-face, via phone or email. Those who express interest in the SFS will receive a Participant Information Sheet and Consent Form (PICF), along with a link to access the SFS Participant Information Video. An Indigenous member of the study staff will then arrange to discuss the study in detail and address any questions, preferably in a face-to-face setting. Potential participants will be advised that participation in the study is entirely voluntary, and individuals can withdraw at any time. The consent process will also explain the nested family support programmes, and parents will have the option to self-refer at any time and allow data linkage with SFS. The written consent of the pregnant participant will include the consent of her infant/young child. Parental/guardian consent may be sought from some young participants (between 16 and 18 years) if deemed necessary by the research team, but only when the youth provides verbal consent to participate in the study. Consent will be obtained in a culturally sensitive manner, including the use of a Participant Information Video recorded by ISC members and the study team, prepared using accessible language to support potential participants who may appreciate support in understanding the complexity of the study.

Participating families will be offered a gesture of appreciation for their time, particularly where an in-person study visit is completed. Incentives may vary across study sites to reflect the unique needs of each community; however, items could include grocery vouchers, pre-paid parking vouchers (if paid parking is needed to access the service), a tote bag and toys. All incentives will be approved by the governing Human Research Ethics and Governance Safety Committee (HREC) prior to being offered to participants.

While full participation in the study is encouraged, some participants may choose to withdraw. Participants who formally withdraw from SFS may request the return or destruction of their biological samples at no personal cost, provided these have not yet been shared with collaborators or used in sub-studies. Following withdrawal from the study, participants will also have the option to either allow the continued use of their previously collected data or request its secure removal from the study dataset.

#### Study regimen

**Figure 2:**
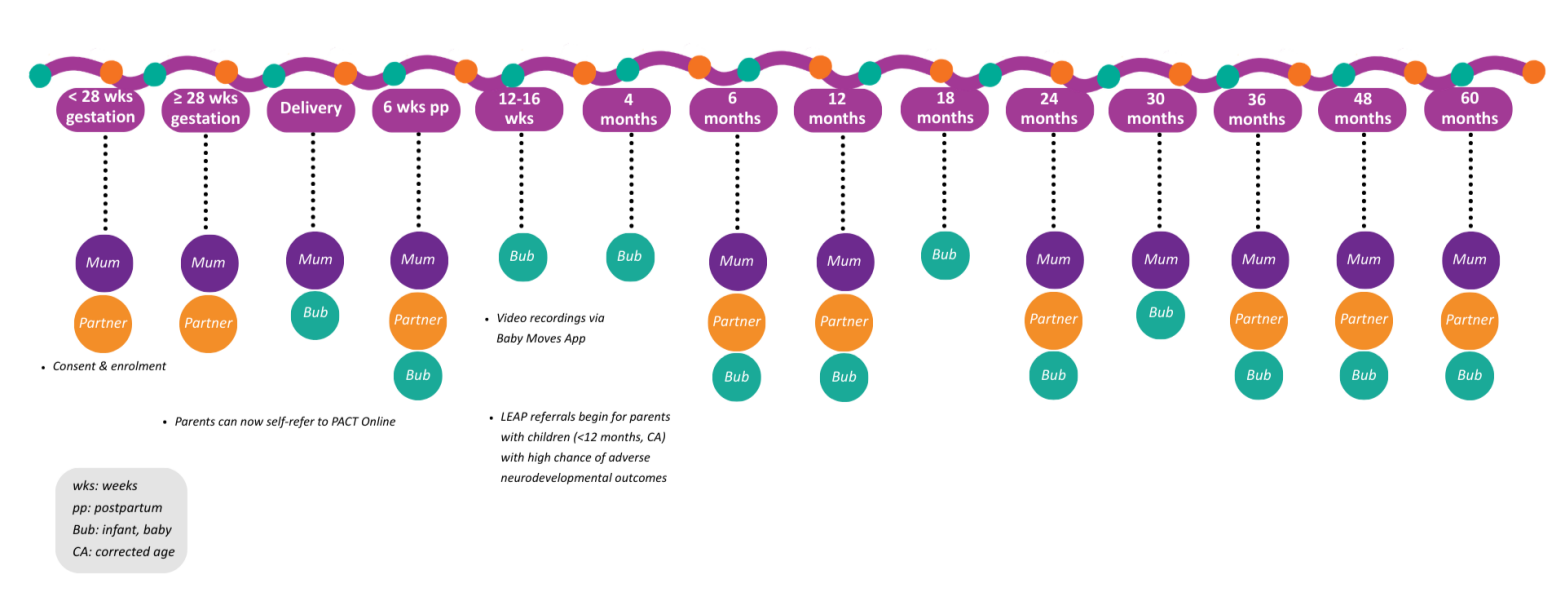
The Strong Families Study recruitment and study visit timeline for participating families. (Note: A detailed description of data collected at each study visit is provided in the online supplemental table 1).

Data will be collected at multiple points throughout the study (figure 2, online supplemental table 1): < 28 weeks gestation, ≥ 28 weeks gestation, at the time of delivery, during the post-delivery period (in the hospital ward), at 6 weeks postpartum and at regular intervals throughout early childhood (from 3 to 36 months CA). Follow-up assessments will continue annually through early childhood.

Following consent, questionnaires will be delivered in person with the help of an Indigenous research assistant or health worker, where possible. A phone option will, however, be made available if requested. In the first two visits, the pregnant participant and, if applicable, their partner complete a series of questionnaires and biological sample collections and undergo initial physical measurements, such as blood pressure and weight. Following birth, the first set of neonatal data is collected, including body measurements and a chart review conducted by a midwife. Subsequent in-person visits will occur throughout the study period, except between 12 and 16 weeks postpartum during which parents are asked to record a 3-min video of their baby’s spontaneous movements for neurodevelopmental assessment (on the General Movements Assessment) (figure 2).^36 37^ Child neurodevelopmental assessments will continue to occur at each visit postbirth. Infants with a confirmed or high chance of a neurodevelopmental disability will be referred to the appropriate family support programmes available within the SFS.^27^,^29^

At the request of the ISC, all study surveys and instruments will be delivered in-person as the first preference using a yarning method, which encourages more informal and relaxed discussions between researchers and participants.^38^,^40^ Furthermore, a health education component will be embedded into the yarns following data collection to increase health literacy and promote healthy behaviours to grow health knowledge in participants of the study. To ensure all team members are appropriately trained and consistent in their approach, yarning guides co-designed by the ISC will be embedded within the electronic data collection forms used for the surveys.

##### Data collection

**Figure 3:**
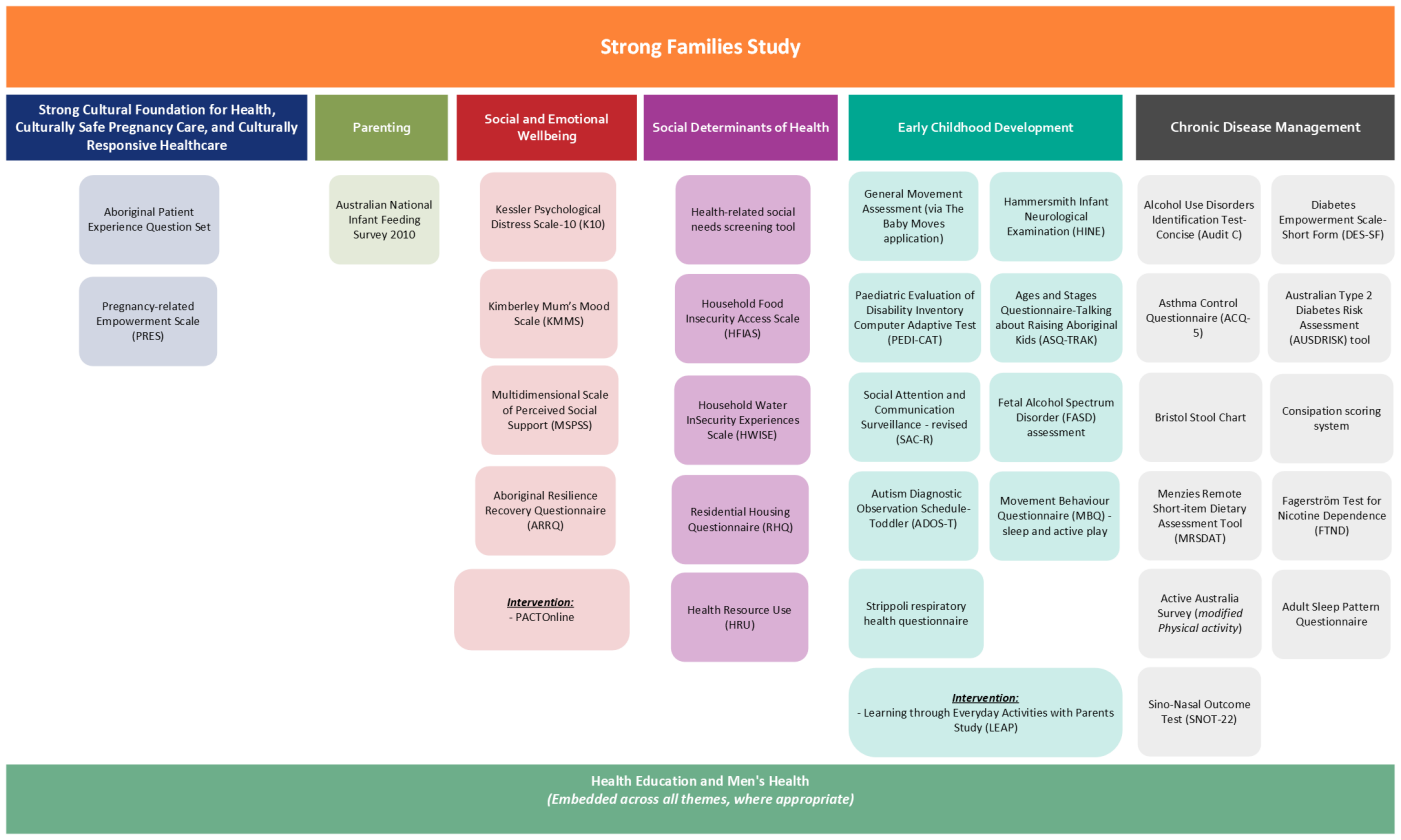
Health themes chosen by the Indigenous Steering Committee and their corresponding questionnaires for participants in the Strong Families Study. (Note: A detailed description of each tool (with citations) is provided in the online supplemental material 1).

The SFS focuses on the prioritised health themes determined through a collaborative co-design process by the ISC. They include social determinants of health; parenting; social and emotional well-being; early childhood development; chronic disease management (particularly focusing on diabetes, cardiovascular disease, bowel care, nutrition, physical exercise and respiratory disease); a strong cultural foundation for health which incorporates culturally safe pregnancy care and culturally responsive healthcare; and health education (figure 3, online supplemental material 1). All themes address health aspects relevant to both men’s and women’s health and will be delivered separately in a culturally appropriate manner.

Biological samples, of blood and urine, along with physical measurements, will be collected throughout the study period. Data collection will also include participant questionnaires and routinely gathered clinical information from medical records, such as medical history, medication usage and antenatal/perinatal data. The survey instruments used in the SFS have received approval from the ISC and will incorporate both validated and culturally appropriate tools, where available (figure 3, online supplemental material 1).

Health education within the SFS is framed not as a data collection theme, but as an act of reciprocity, providing meaningful knowledge and support back to the community across the various health themes. This will be embedded throughout the study as well as a referral to the two nested family support programmes. The ISC has requested that health education be delivered by the research team during each study visit, connected to the purpose of various research themes and data surveys. This will increase the health literacy of families in the study. Health education scripts have been developed for all research staff, and the ISC has reviewed and approved these.

A parenting support programme will be offered that focuses on social and emotional well-being called PACT Online, grounded in Acceptance and Commitment Therapy. It has been tested with parents of children diagnosed with or with a high likelihood of neurodevelopmental disability or developmental delay and was designed to be flexible and appropriate to supporting all parents.^27^ This programme is directly offered to parents at 3 months CA, but families can self-refer at any point during the study (figure 2, online supplemental figure 1). Within this study, a First Nations PACT Online package will be codesigned. For infants aged <12 months CA in the study who have a high chance of adverse neurodevelopmental disability including cerebral palsy, autism or fetal alcohol spectrum disorder, a referral will be offered to families to participate in the Learning through Everyday Activities with Parents Study (LEAP) (figure 2, online supplemental figure 1). LEAP is a randomised early support trial delivered in the home by Indigenous family support workers. Compared with a health advice programme, it includes goal-directed skill training, responsive parenting, environmental enrichment through learning games and parent educational modules.^28 29^

At regular intervals, data linkage between the SFS database and the LEAP and PACT Online teams will take place to enable analysis of the family support programmes within the context of the SFS study. To ensure accurate referral processes, parents’ samples and questionnaires will be regularly reviewed for signs of clinically relevant findings or having an increased chance of developing a clinically relevant disease (see online supplemental figure 1 on flow chart for infant testing and referral processes in the SFS). Where family support programmes are required but outside the scope of the study, access to services, including liaising with the primary care team, will be facilitated by the study team.

To support retention over the 5-year follow-up, we will prioritise in-person, culturally safe data collection using a yarning approach, supported by flexible scheduling, telehealth or phone options, and alignment with existing antenatal or paediatric appointments. Retention will be strengthened through sustained, trusting relationships fostered by ACCHOs and partnering hospitals; regular personalised contact (SMS, calls, emails and periodic check-ins); and routine updates to contact details, including alternative contacts. Reminder messages will be sent for upcoming visits, and site-approved appreciation or incentives will be offered where appropriate. We will also create a positive participant experience by ensuring visits are welcoming and culturally respectful and by acknowledging family and child milestones to support ongoing engagement.

##### Sample size and statistical power

The sample size of 400 families followed prospectively was chosen to provide sufficient power to examine associations between perinatal strengths/risk factors and pregnancy, birth and early childhood outcomes. Assuming approximately 5% of the cohort will have the primary outcome of ‘chance of cerebral palsy/autism spectrum disorder/neurodevelopmental outcome’, this sample gives 80% power (α=0.05) to detect absolute differences of ≥8 percentage points between binary risk factor groups. For more common binary outcomes, differences of >28% can be detected. For continuous outcomes, we will be able to identify between-category differences of >0.3 SD (Z-score units). These estimates are conservative, as repeated measurements over time and mixed-effects models will further improve precision.

##### Statistical analysis

To examine relationships between perinatal strengths/risk factors and continuous outcomes (eg, Kimberley Mum's Mood Scale (KMMS)), we will use linear or mixed effects regression for repeated measures. Binary outcomes will be analysed using logistic regression, and count outcomes with Poisson or other suitable count models. Analyses will account for the multi-site teletrial design by adjusting for clustering at the site/community level (eg, through random or fixed effects or robust standard errors). For multivariable modelling, candidate predictors will be screened individually, with final model selection guided by Bayesian Information Criteria. Model calibration will be checked graphically, and internal validation performed via bootstrap resampling.

Missing data will be treated case-by-case using multiple imputation when data are plausibly missing at random, and pattern mixture methods with sensitivity analyses when not missing at random mechanisms are likely. Longitudinal mixed-effects models will use all available observations under missing at random assumptions. If differential loss to follow-up occurs, we will apply weighting-based sensitivity analyses (eg, inverse probability of follow-up weighting). Each analysis will include appropriate adjustments for multiple comparisons to balance type I and II error risks.

For the PACT Online and LEAP programmes, effectiveness will be investigated using mixed effects models with programme participation included as the main effect and models adjusted for baseline factors. Differences in characteristics of families who do and do not participate in the programmes will be assessed. If any differences exist, they will be adjusted for in analyses using weighting methods such as propensity matching. Cohort embedded summaries of eligibility, referral and uptake will be reported descriptively. The sensitivity of observational comparisons (eg, uptake vs non-uptake) will be assessed.

An implementation and evaluation analysis will be undertaken and reviewed by the ISC. The RE-AIM framework will be applied to assess the reach, effectiveness, adoption, implementation and maintenance of SFS across all the study sites.^41^ A combination of quantitative metrics (eg, engagement numbers, training outcomes) and qualitative insights from interviews and focus groups will be used. The evaluation will draw on study monitoring data, staff training assessments, participant surveys and yarning interviews to measure how effectively the study is implemented, delivered and taken up. This process ensures comprehensive staff training, strategic resource allocation and identification of site-specific needs while maintaining consistent adherence to the study protocol. Findings for each RE-AIM domain will be summarised using descriptive quantitative indicators and thematic analysis of qualitative data.

### Participant confidentiality and safety

Pre-screening lists will be stored on secure, password-protected drives accessible only to the research study team and will be permanently deleted according to the data management plan. In line with protecting participant confidentiality, databases will be password protected on restricted access services. Hard copy and soft copy files will be kept for 33 years after completion of the study. This is in accordance with the University of Queensland-Mater Research Data Collection, Entry, Storage, Movement and Destruction policy guided by prevailing standards for Australian clinical trial research and national legislation.

In the event of unexpected findings or urgent care needs, participant welfare will be the priority, with safeguarding protocols in place at all sites. Additionally, referral pathways are established for participants experiencing adverse health needs including mental health outcomes to ensure timely and appropriate support.

### Data management

Data will be collected using electronic forms and entered into a secure, web-based software platform, Research Electronic Data Capture (REDCap),^42^ hosted at the University of Queensland and backed up periodically to protect the data. At each study site, data collection and entry will be carried out by experienced and qualified research midwives and Indigenous research assistants with appropriate access to REDCap. The quality and validity of data will be ensured by implementing rigorous data verification procedures, including regular audits, double data entry checks and adherence to the SFS protocol.

### Indigenous data sovereignty

The ISC and lead research team have developed a shared understanding of the Indigenous data sovereignty principles that underpin the study, which are based on those of the Maiam Nayri Wingara Indigenous Data Sovereignty Collective.^43^ Primarily, these principles are driven by the self-determination of Indigenous people to own and undertake all decisions in data aims, data collection and data management. Additionally, in the SFS, data collection is limited to information with a defined purpose, as determined by the ISC, acting as a proxy for their communities. As previously mentioned, the study acts solely as data custodian, and ownership of the data remains with the individual participant, who may request the destruction or repatriation (in the case of biological samples) or removal from the dataset at any time. As previously highlighted, the ISC committee membership will be reviewed annually to ensure appropriate governance remains in place over the term of the data custodianship.

De-identified data for the Indigenous community will be returned to community partners at regular intervals throughout the study lifespan, following a schedule agreed on with each site/region or on request. This approach ensures that services can continue to adapt and align their health services with the evolving health needs and priorities identified by participants from their communities. Data management and policy will be reviewed annually, along with the Terms of Reference for the ISC, to maintain ongoing transparency with the committee. Any request to use data or samples from the study beyond previously established and agreed uses will require prior approval from the ISC before ethical approvals are sought.

## Discussion

The Strong Families Study focuses on Indigenous families in Queensland, Australia. It will track the health and well-being of Indigenous families from pregnancy to early childhood (figure 2), incorporating nested family support programmes designed to strengthen families’ capacity and self-determination. Notably, it is the first study of its kind to be co-designed with Indigenous communities and to actively include both Indigenous parents in its recruitment process. Central to the study’s design is the integration of an Indigenous Governance framework, ensuring that the research is co-led, culturally responsive and community-driven. The included surveys were chosen by the ISC over a broad range of health-related themes, providing a comprehensive understanding of the health and needs within Indigenous communities.

A key strength of this study is the multidisciplinary nature of the study’s research team. The diverse expertise within the team, encompassing fields such as epidemiology, psychosocial health, perinatal health, chronic disease, paediatrics and Indigenous knowledge systems, enhances the breadth of the study design. This multidisciplinary approach, bolstered by a strong number of senior Indigenous academics, fosters a holistic understanding of the birth cohort study, ensuring that various dimensions of health, well-being and culture are incorporated. Also, the early and sustained involvement of the ISC represents a significant asset to the study design process. Globally, consumer-driven research is becoming widely adopted,^44 45^ particularly in Australia with the support of the large health research councils such as the National Health and Medical Research Council (NHMRC),^46^ the Medical Research Future Fund (MRFF)^47^ and the Australian Research Council (ARC).^48^ The active involvement of ISC members in leadership and throughout the co-design process strengthens the study’s cultural safety and acceptability and also promotes co-ownership of the project. This co-design approach is essential for ensuring that SFS research outcomes align with the needs and priorities of Indigenous communities around Queensland. Such community involvement fosters mutual trust, enhances the cultural relevance of the research and provides valuable benefits for both researchers and the community.^49^

In the spirit of reciprocity,^46^ SFS staff embedded within communities will play a central role in building local capacity through upskilling Indigenous healthcare workers to identify neurodevelopmental disabilities early and help facilitate timely family support programmes. As highlighted by the ISC, SFS is grounded in a strength-based approach that prioritises community expertise and leadership. In nurturing local knowledge and skills, this approach fosters long-term sustainability, empowering communities to respond effectively to their own needs. In Australia, Aboriginal Participatory Action Research that is deeply embedded in community has been shown to empower Indigenous people; enhance existing services, for example, mainstream health services; and promote meaningful knowledge translation.^19 50 51^ Importantly, findings from SFS will be communicated directly back to communities. This aligns with SFS’s commitment to reciprocity and supports the development of policy and healthcare decisions that are both region-specific and responsive.

The inclusion of both partners in the SFS study represents an inclusive and holistic aspect of the birth cohort study, particularly in Indigenous health research. This approach addresses a notable gap in the existing literature, where the perspectives and health outcomes of both Indigenous partners are often under-represented. By incorporating their voices, the study contributes to a broader understanding of family health and dynamics. A further strength of the study lies in its broad measurement of health themes using validated and culturally appropriate tools. The addition of a wide range of health indicators spanning from pregnancy to early childhood enhances the study’s capacity to capture the multifaceted nature of health and well-being. The use of culturally appropriate and validated instruments chosen by the ISC strengthens the methodological rigour of the study, ensuring that the data collected are scientifically robust and also inclusive and culturally sensitive.

The collection of biological samples represents a unique aspect of the study. To the best of our knowledge, this will be the first birth cohort study to collect and analyse such biological parameters within the two-parent-and-child triad across multiple regions in the state of Queensland. This novel component provides valuable opportunities to better understand the interplay between biological, environmental and social determinants of health, offering deeper insights into the health trajectories of Indigenous families.

There are several potential limitations of the study. As with other longitudinal studies, the Strong Families Study involves significant time and resource investment for its rigorous design and implementation. For participants, particularly those with young children, the requirement for multiple in-person visits to the study site could be burdensome. This may lead to fatigue or disengagement, thereby impacting participant retention and data consistency over time. Moreover, the need for consistency in data collection, delivery of yarning scripts, health education delivery and adherence to the study protocol across different locations requires careful planning and resource allocation. The study has proactively established a rigorous implementation and evaluation process using the RE-AIM framework.^41^ By implementing these processes, the study aims to mitigate potential challenges, adapt study procedures to the unique contexts of each community and enhance the overall rigour and reliability of the research outcomes. A further limitation is the restriction of participant enrolment to individuals who are <28 weeks gestation. This criterion may exclude late presenters, potentially reducing the generalisability of the findings to the broader population.

### Ethics and dissemination

Letters of endorsement were obtained from the study site partner services across Queensland and submitted as part of the ethics process. The Strong Families Study was approved by the Mater Misericordia Limited Human Research Ethics and Governance Safety Committee (HREC/MML/105191) and ratified by the University of Queensland Human Research Ethics Committee (2025/HE001924). The nested family support programmes embedded in the SFS were approved as follows: LEAP - (HREC approvals: HREC/20/QCHQ/63906, HREC/2019/QCH/50533, HREC/QTHS/56008 and The University of Queensland Human Ethics Research Committee (2020000185)) and PACT Online - (023/HE000040). The co-design process, which includes community consultation through the ISC, was approved as part of the I-Priorities study (HREC/MML/72562) and ratified by the University of Queensland Human Research Ethics Committee (2022/HEO01885). SFS findings will be shared with the Australian Institute of Aboriginal and Torres Strait Islander Studies data repositories and disseminated to the scientific community, government agencies, funding bodies, research institutes and participating health services to inform policy. Study snapshots will be provided to interested participants through newsletters via email or postal mail.

## Supplementary material

10.1136/bmjopen-2025-113766 Supplementary file 1

## Data Availability

Access to the data may be granted on a limited basis to external investigators, contingent on approval from the ISC and the CEC and with appropriate ethical clearance. All requests must demonstrate alignment with the study’s objectives and uphold the principles of Indigenous data sovereignty.
