# Supplementary material for: Strong Families Study: protocol for a co-designed birth cohort study with Aboriginal and Torres Strait Islander families in Queensland, Australia
Source: BMJ Open. 2026 May 28;16(5):e113766. doi: 10.1136/bmjopen-2025-113766 (PMC13223655; doi:10.1136/bmjopen-2025-113766)
Supplement: Supplementary file 1 [file bmjopen-16-5-s001.docx]

# **Supplementary Material:**

**The Strong Families Study: Protocol for a Co-designed Birth Cohort Study with Aboriginal and Torres Strait Islander Families in Queensland, Australia**

# **Authors:**

Salma Mohamed Ahmed^+1^ and Emily S. Dorey^+1^, Davina Smith*^1^, Loretta Weatherall*^1^, Rhiannon Friday*^1^, Luciana F. Massi^1^, Rebecca Rooney^1^, Diana Hermith-Ramirez^2,3^, Emma Kendall^1^, Kai Wheeler*^1,4^, Anne-Marie Eades*^5^, Maree Toombs*^6^, Roslyn N. Boyd^2,4^, Rhonda Marriott*^7^, Sandra J Eades*^8^, Katherine Benfer^2,4^, Natasha Reid^4^, Koa Whittingham^2,4^, Robert S. Ware^3,9^, Elizabeth Martin^4,10^, Paul D. Robinson^11^, Vicki Clifton^4,12^, Leonie Kaye Callaway^4,13^, Sailesh Kumar^4,14^ , and Kym M. Rae^1,4 x^, on behalf of the Strong Families Indigenous Steering Committee*^1^.

**Authors proudly identify as Aboriginal and/or Torres Strait Islander*

*^+^Co-first Author*

# **Affiliation:**

1. Indigenous Health Research Group, Mater Research Institute - The University of Queensland, QLD, Australia
2. Queensland Cerebral Palsy and Rehabilitation Research Centre, Child Health Research Centre, Faculty of Health, Medicine and Behavioural Sciences, The University of Queensland, QLD, Australia
3. Griffith University, QLD, Australia
4. Faculty of Health, Medicine and Behavioural Sciences, The University of Queensland (UQ), QLD, Australia
5. Curtin School of Nursing, Curtin University, WA, Australia
6. Faculty of Medicine and Health, University of New South Wales, NSW, Australia
7. Ngangk Yira Centre for Change, Murdoch University, WA, Australia
8. Melbourne School of Population and Global Health, University of Melbourne, VIC, Australia
9. Wesley Research Institute, QLD, Australia
10. Duke-NUS Medical School, Singapore
11. Children’s Health and Environment Program, Child Health Research Centre, University of Queensland, QLD, Australia
12. Mater Research Institute - The University of Queensland, QLD, Australia
13. Women’s and Newborn Services, Royal Brisbane and Women’s Hospital, Metro North Health, QLD, Australia
14. Mater Medical Research Institute - Maternal & Fetal Medicine, QLD, Australia

**^x^ Corresponding author:**

Professor Kym M. Rae

Address: Mater Research Institute - UQ,

Level 3, Aubigny Place,

South Brisbane, QLD, 4101

AUSTRALIA

Email: [kym.rae@uq.edu.au](mailto:kym.rae@uq.edu.au)

## **Supplementary Method:**

The following supplementary method provides additional information on the SFS themes prioritised by the ISC with the corresponding measurement tools/surveys:

### **Social and Emotional Wellbeing**

This theme assesses the mental health and wellbeing of SFS families via:

- *Kessler Psychological Distress Scale-10 (K10):* a 10-item screening tool for identifying levels of distress in individuals in the past month.^1^ It is a 5-point Likert scale yielding scores from 10 to 50, with higher scores representing higher distress level, including anxiety and/or depression.
- *Kimberley Mum’s Mood Scale (KMMS):* a culturally tailored tool for detecting perinatal depression and anxiety in Indigenous Australian women.^2^ Part one of the tool includes 10 questions based on the Edinburgh Postnatal Depression Scale (EPDS)^3^ using a visual Likert scale, with scores of 9 or above indicating elevated risk. For question 10, any score above zero requires further enquiry. Part two of the tool encourages a narrative exploration of responses from Part one by exploring the following themes: support, major stressors, self-esteem/anxiety, relationships, childhood experiences, substance misuse, and social, emotional and cultural wellbeing.^2^
- *Multidimensional Scale of Perceived Social Support (MSPSS):* a 12-item scale assessing perceived social support from the following sub-scales: (i) family, (ii) friends, and (iii) significant others.^4^ Items are rated on a 7-point Likert scale, with higher scores indicating greater social support. A total score is calculated as the sum of all items (ranging from 12 to 84), while the sub-scale scores are calculated by averaging the four items per category.
- *Aboriginal Resilience Recovery Questionnaire (ARRQ):* a 60-item multidimensional tool that assesses resilience, healing and recovery in Indigenous populations on a 5-point Likert scale.^5-7^ This strength-based tool focuses on the following domains: i) cultural, community and relationship strengths and resources (questions 1 to 21), ii) personal strengths and resources (22 to 50), and iii) single item questions on additional strengths and resources (51 to 60). A total strengths score is calculated based on the sum of the first two domains only, with higher scores indicating stronger protective factors and greater resilience.

### **Social Determinants of Health**

This theme focuses on environmental, economic and social factors influencing health outcomes:

- *Residential Housing Questionnaire*: a 16-item survey assessing current housing and environmental conditions.^8^
- *Health-related social needs screening tool*: identifies an individual’s needs in areas such as living situation, food situation, transportation and utilities.^9^ Following the recommendations from the ISC, only questions 1 to 6 are included in the SFS protocol.
- *Household Food Insecurity Access Scale (HFIAS):* a 9-item tool measuring the severity of food insecurity in a household over the past month.^10^ Responses are rated on a 3-point Likert scale and items are summed to yield a total score ranging from 0 to 27. A higher score indicates a greater severity of food insecurity.
- *Household Water InSecurity Experiences Scale (HWISE):* a 12-item tool capturing water-related challenges at the household level over the past month.^11^ Scored on a 4-point Likert scale, a total score is calculated ranging from 0 to 36, with a higher score reflecting greater household water insecurity or a threshold score of 12 or above indicating potential concern.
- *Health Resource Use (HRU):* a tailored SFS questionnaire to capture healthcare use by Indigenous peoples over a six month period, including engagement with both mainstream services and traditional or alternative medicine.

### **Parenting**

This theme focuses on the caregiving practices, particularly around infant feeding and nutrition:

- *Australian National Infant Feeding Survey (ANIFS) 2010*: collects information on breastfeeding intentions, baby’s first feed, breastfeeding habits, infant feeding, including introduction to solid foods.^12^

### **Early Childhood Development**

This theme pertains to developmental milestones and early neurodevelopmental health of children which will be completed by either the parent or a trained research staff member:

- *General Movement Assessment (via The Baby Moves application (app)):* a smartphone app used to assess neurodevelopmental outcomes using video recording of infants between 3 and 4 months’ corrected age.^13^ The videos are then assessed by a trained assessor of the GMA and scored according to Prechtl’s GMA.^14^ Fidgety movements will be classified as: normal if they are consistently present, absent if they are missing or only appear occasionally, and abnormal if they are unusually fast or exaggerated.^15, 16^
- *Ages and Stages Questionnaire-Talking about Raising Aboriginal Kids (ASQ-TRAK):* a cross-cultural adaptation of the standard ASQ-3 for identifying Aboriginal children with developmental delays in a culturally respectful and appropriate way.^17, 18^ The tool encompasses the following five domains: communication, gross motor, fine motor, problem-solving, and personal-social, comprising a total of 30 questions (six items per domain). Each domain item is scored from 0 to 10 (“Yes”= 10, “Sometimes”= 5, “Not yet”= 0). A total score (sum of each item) for each domain is obtained, ranging from 0 to 60. Total domain scores are compared with the established cut-off point for the age group, indicating whether the child is at risk of developmental delay.
- *Hammersmith Infant Neurological Examination (HINE*): a standardised tool used to screen for early signs of neurological disorders, particularly cerebral palsy, in infants aged 2 to 24 months.^19^ The tool includes 26 items across the following domains: cranial nerve function, posture, movements, muscle tone and reflexes. Each item is scored from 0 (severely abnormal) to 3 (normal), with a maximum total score of 78.^19, 20^ Lower total scores indicate a higher risk of developmental disorders, with age-specific cut-off points applied.
- *Paediatric Evaluation of Disability Inventory Computer Adaptive Test (PEDI-CAT):* a validated, standardised assessment tool completed by parents to evaluate children (across 21 age groups) with physical and/or behavioural challenges.^21-23^ It includes the following domains: daily activities (68 items), mobility (97 items), social/cognitive function (60 items) and responsibility (only ≥ 3 years old) (51 items). The first three functional domains can be rated using a 5-point Likert Scale, i.e ‘easy’, ‘a little hard’, ‘hard’, ‘unable’ or ‘I don’t know’. The responsibility domain uses a separate 5-point Likert scale, ranging from ‘adult/caregiver has full responsibility’ to ‘child takes full responsibility without any directions’. The tool provides a normative standard score (expressed as a T-score and age percentile range, such as <5th or 5th–25th percentile) and a scaled score, which is recommended for tracking changes over time.^21, 22^
- *Autism Diagnostic Observation Schedule- Toddler (ADOS-T):* a standardised tool for young children aged 12 to 30 months suspected of having autism spectrum disorder.^24^ The tool assesses social engagement, communication, and play, and includes eleven activities. By summing specific item scores, a classification is generated that indicates whether an individual is likely to be on the autism spectrum, has Autism Spectrum Disorder (ASD) or falls within non-spectrum conditions. This classification can then support clinicians or researchers as one component of a broader diagnostic assessment.^24^
- *Fetal Alcohol* *Spectrum Disorder (FASD) assessment*: involves photographs and/or clinical measurements of the child if FASD is suspected.^25^ Facial photographs are taken in three views: frontal, three-quarter, and lateral, using a digital camera. These images are used to show the palpebral fissures, upper lip, and philtrum.^25^ The photographs are then uploaded into the FAS Facial Photographic Analysis Software (Version 2.1.0).^25, 26^ Once the facial features are measured, the software generates a report. The results are presented using a 4-point Likert scale: Rank 1 indicates no FAS facial features, Rank 2 indicates mild features, Rank 3 indicates moderate features, and Rank 4 indicates severe FAS facial features.^25-27^
- *Sleep section of Movement Behaviour Questionnaire:* this is a validated assessment tool used to assess sleep patterns, habits and quality of children in the past week.^28^ Based on the guidelines, children under 3 years should have at least 660 minutes (11 hours) of sleep per day, while those aged 3 years and older should have at least 600 minutes (10 hours) per day.
- *Strippoli respiratory health questionnaire*: This parent-completed questionnaire assesses respiratory symptoms and related conditions in 1-year-old children over the past 12 months.^29^ It specifically includes items on wheezing, coughing, medical diagnoses, treatments, ear, nose and throat symptoms, family history, and the household and environmental context.
- *Active play section of the Movement Behaviour Questionnaire (MBQ*): this is a validated assessment tool used to evaluate physical activity (PA) levels of children over the past week using four questions.^28^ A child is meeting the guidelines if they meet at least three hours of total PA per day, including 1 hour of energetic play.
- *Social Attention and Communication Surveillance* - revised (SACs-R): a surveillance tool used for early identification of ASD.^30, 31^ It comprises 12 to 15 early social-communication behaviours observed at age range 11-30 months. It has five key items that are most predictive of a later autism diagnosis. Children who display atypical behaviours on at least three of these items are considered at high likelihood for autism and referred for further assessment

### **Chronic Disease Management**

This theme focuses on risk, symptoms and behaviours related to chronic conditions in SFS adults:

- *Alcohol Use Disorders Identification Test-Concise (Audit C):* a 3-item tool for identifying risky alcohol use using a 5-point Likert scale, with each response scored from 0 to 4, for a total score range of 0 to 12.^32, 33^ A score of 3 or more in women, and a score of 4 or more in men, indicates hazardous drinking or a possible alcohol use disorder.
- *Asthma Control Questionnaire (ACQ-5):* assesses asthma symptoms over the past week in people with asthma using five questions rated on a 7-point Likert scale (from 0 = no impairment to 6 = maximum impairment).^34^ The final score is the average of the 5 questions, and results are interpreted as follows: ≤ 0.75 (well controlled), 0.75 to 1.5 (partially controlled), and >1.5 (not well controlled).
- *Australian Type 2 Diabetes Risk Assessment (AUSDRISK) tool*: a screening tool used to estimate the risk of developing Type 2 diabetes using 10 questions, each with points.^35, 36^ Scores ≤ 5 mean low risk of developing diabetes, 6 to 11 refers to intermediate risk, and ≥ 12 refers to high risk.
- *Bristol Stool Chart and Constipation scoring system*: Bristol Stool Chart assesses bowel health using a visual scale to classify stool form.^37^ The latter measures symptoms of constipation based on 8 questions. The score is out of 30 with >15 points indicating constipation.^38^
- *Diabetes Empowerment Scale-Short Form (DES-SF):* a 8-item validated tool used to evaluate psychosocial self-efficacy in managing diabetes on a 5-point Likert scale.^39, 40^ The total score is the average score of all items, with a higher value representing higher perceptions of psychosocial self-efficacy.
- *Fagerström Test for Nicotine Dependence (FTND):* a validated tool for measuring the intensity of nicotine addiction among cigarette smokers based on six questions.^41^ Scores range from 0 to 10, with higher scores indicating greater dependence. The tool also includes an additional six questions on vaping with a similar scoring system to that of nicotine.^42^
- *Active Australia Survey (modified Physical activity):* captures the frequency, duration, and intensity of adult physical activity in the preceding week.^43, 44^ The physical activity score is derived by summing the total weekly minutes of activity, weighted by the metabolic equivalent (MET) values assigned to each category: (walking minutes × 3.33 METs) + (moderate-intensity activity minutes × 3.33 METs) + (vigorous-intensity activity minutes × 6.66 METs). Australian adults are recommended to achieve at least 600 MET-minutes of physical activity per week,^45^ whereas for pregnant women, the threshold is slightly lower at 500 MET-minutes per week.^44^ In addition, sedentary behaviour is captured using a modified version of the International Physical Activity Questionnaire (IPAQ).^46^ Average daily sitting time (hours) is calculated using a weighted formula to account for differences between weekdays and weekends: ((weekday sitting time × 5) + (weekend sitting time × 2) / 7). A threshold of ≥ 8 hours per day is used to classify poor sedentary behaviour.^47^
- *Menzies Remote Short-item Dietary Assessment Tool (MRSDAT):* an online food frequency tool that was developed with and for remote Aboriginal populations.^48, 49^ It comprises 32 questions across different food groups (including traditional foods) and drinks consumed. It auto-calculates the Dietary Guideline Index score (0-100), measuring the degree of adherence of the reported diet to the Australian Dietary Guidelines.^48^
- *Adult Sleep Pattern Questionnaire (ASPQ):* a validated self-report questionnaire used to assess sleep quality over the past week.^50-53^ The tool captures sleeping patterns, sleep-related impairment, sleep-wake behaviours, sleep beliefs and Insomnia Severity Index (in the past two weeks).
- *Sino-Nasal Outcome Test (SNOT-22):* this validated tool measures symptoms and quality of life impact of chronic rhinosinusitis based on 22 questions using a 6-point Likert scale.^54^ The total score ranges from 0 to 110, with higher scores indicating a greater impact on quality of life.

### **Strong Cultural Foundation for Health, Culturally Safe Pregnancy Care, and Culturally Responsive Healthcare:**

These three interconnected themes explore the experiences of Indigenous people with the broader health services, including the use and delivery of culturally safe and responsive healthcare practices via:

- *Aboriginal Patient Experience Question Set*: a 31-item tool that measures the experiences and satisfaction of Aboriginal patients with healthcare services.^55, 56^ Tool covers areas such as experience with doctors, nurses, treatment, hospital experience including support.
- *Pregnancy-related Empowerment Scale (PRES):* a validated tool that measures a pregnant person’s sense of empowerment during pregnancy using 16 questions on a Likert scale (1: strongly disagree to 4: strongly agree).^57^ The domains included provider connectedness, skilful decision-making, peer connectedness and gaining voice. The mean score is calculated from a total possible score of 64, with higher scores reflecting a more positive perception of pregnancy-related empowerment.

## **Other components of SFS:**

### *Health education:*

Across all health themes, health education will be integrated and delivered to participants by the Aboriginal Health Workers. Research staff will receive comprehensive training on the delivery of this education through various methods, including resource manuals for each tool, face-to-face training, and other blended training approaches across each study site.

### *Clinical measurements:*

1. Anthropometry of parents (weight, height, skinfold thickness, waist circumference): These measures will be taken for each participant across the study.
2. Blood pressure and heart rate
3. Infant anthropometry: infant weight, length, head circumference, abdominal circumference, mid-upper arm circumference
4. FASD images: as previously described, this involves photographs of facial features for children suspected of FASD.^25, 27^

### *Sociodemographic questionnaires:*

Demographics: age, date of birth, marital status, country of birth, Indigenous identity, education, income, employment, residential address, gestation at enrolment, estimated due date, main language spoken, health status, Medicare eligibility, private health insurance coverage, among others.

### *Biological samples:*

Urine and blood samples will be collected from family members at specific study visits (Supplementary Table 1).

### *Data Linkage:*

Data linkage downloads will be completed annually for the following key datasets:

1. Mater Medical Records: clinical data from the Mater Health system with information on routine data of participants.
2. Medicare Benefits Schedule (MBS): a program that provides subsidised health services by the Australian Government under Medicare, with data on medical services and procedures used.
3. Pharmaceutical Benefits Scheme (PBS): provides data on prescribed medications that are subsidised by the Australian Government.
4. National Assessment Program – Literacy and Numeracy (NAPLAN): a standardised nationwide program administered to children in Years 3, 5, 7 and 9 across five academic areas (i.e., writing, reading, grammar and punctuation, numeracy and spelling).^58^
5. Australian Early Development Census (AEDC): administered nationally to children in their first year of full-time school at ages 5-6 years every three years.^59^ The purpose is to provide a snapshot of early childhood development across Australia.
6. LEAP and PACT Online study outcomes: the results of participants referred to both the LEAP^15, 60^ and PACT Online^61^ programs will be linked through data integration processes to enable evaluation of health outcomes.
7. Queensland Health: clinical, administrative, and public health data relevant to a patient's care can be accessed through Queensland Health systems.
8. International Classification of Diseases (ICD) codes: used to systematically identify and classify health conditions. They will facilitate linkage between clinical data and study records, enhancing the accuracy and consistency of health information.

# **Supplementary Table 1**: SFS questionnaires, measures and biological samples completed by participants throughout the study period

| **Visit** | **Pregnant Participant** | **Partner** | **Baby** |
| --- | --- | --- | --- |
| **< 28 weeks gestation** | ***Questionnaires:*** Consent form, ARRQ, MRSDAT, Audit C, ACQ, FTND, HFIAS, HWISE, HRU, Health-related social needs, KMMS, Medical history, MSPSS, PRES, RHQ  ***Samples/measures:*** Blood, Urine, BP, HR, Weight, Height, Skinfold thickness | ***Questionnaires:*** Consent form, ACQ, ARRQ, Audit C, AUSDRISK, MRSDAT, FTND, HFIAS, HWISE, HRU, Health-related social needs, Medical history, MSPSS, RHQ  **Samples*/measures*:** Same as pregnant participant, and waist circumference | None |
| **≥ 28 weeks gestation** | ***Questionnaires:*** ACQ, Audit C, DES-SF, ANIFS, HFIAS, HWISE, FTND, KMMS, MRSDAT, PRES, RHQ, HRU, Health-related social needs, Medical history, SNOT-22, Physical activity  ***Samples/measures:*** BP, HR, Weight, Height, Skin fold thickness | ***Questionnaires:*** ACQ, ARRQ, Audit C, AUSDRISK, FTND, MRSDAT, RHQ, HFIAS, HWISE, HRU, Health-related social needs, Medical history, SNOT-22, Physical activity  ***Samples/measures***: BP, HR, Weight, Height, Skin fold thickness, Waist circumference | None |
| **Delivery/On ward** | ***Questionnaires:*** Chart review (of during labour and delivery), Delivery details, Medications used | None | ***Questionnaires:*** Chart review, Delivery details, Medications used  ***Samples/measures:*** Guthrie heel prick, Body measurements |
| **6 weeks postpartum** | ***Questionnaires:*** Aboriginal patient experience question set, ACQ, ASPQ, Bristol stool chart, Constipation score, HFIAS, HWISE, FTND, KMMS, MRSDAT, Physical activity, SNOT-22, HRU, RHQ, Medical history  ***Measures*:** BP, HR | ***Questionnaires:*** Aboriginal patient experience question set, ACQ, ASPQ, Bristol stool chart, Constipation score, HFIAS, HWISE, FTND, K10, MRSDAT, Physical activity, SNOT-22, HRU, RHQ, Medical history, Health-related social needs, ARRQ, Audit C  ***Measures:*** BP, HR | ***Questionnaires:*** ANIFS, Sleep pattern (MBQ), Bristol stool chart, Constipation score  ***Samples/measures:*** Lung function test, BP, HR, Body measurements including length, weight |
| **12-16 weeks postpartum** | None | None | ***Video***: Baby Moves App (2 short videos) |
| **4 months** | None | None | ***Questionnaires:*** Bristol stool chart, Constipation score, Medical history, Sleep pattern (MBQ)  ***Measures:*** Length, Weight, BP, HR |
| **6 months** | ***Questionnaires:*** ARRQ, DES-SF, FTND, K10  ***Samples/measures:*** Blood, Urine, BP, Weight, Height | ***Questionnaires:*** DES-SF, FTND, K10  ***Samples/measures:*** None | Questionnaires: HINE, ANIFS, ASQ-TRAK, Bristol stool chart, Constipation score, Medical history, Sleep pattern (MBQ)  ***Samples/measures:*** Weight, Length |
| **12 months** | ***Questionnaires:*** ARRQ, ACQ, ASPQ, AUSDRISK, Bristol stool chart, Constipation score, HFIAS, HWISE, FTND, Health-related social needs, HRU, K10, Medical history, MRSDAT, MSPSS, Physical activity, RHQ, SNOT-22  ***Samples/measures:*** Blood, Urine, BP, HR, Weight, Height | Same as Pregnant participant | ***Questionnaires:*** HINE, ASQ-TRAK, ANIFS, Bristol stool chart, Constipation score, Strippoli respiratory health questionnaire, HRU, PEDI-CAT, SACs-R  ***Samples/measures:*** Lung function test, BP, HR, Weight, Height, FASD image (if high risk) |
| **18 months** | None | None | ***Questionnaires:*** ANIFS, Bristol stool chart, Constipation score, Medical history  ***Measures***: BP, HR, Weight, Height |
| **24 months** | Same as 12 months, (not including ARRQ) | Same as 12 months | ***Questionnaires:*** ASQ-TRAK, ADOS-T (if indicated), Bristol stool chart, Constipation score, HINE, PEDI-CAT, Active play (MBQ), Strippoli respiratory health questionnaire, SACs-R, Medical history, Sleep pattern (MBQ), HRU  ***Samples/measures:*** Lung function test, BP, HR, Weight, Height, FASD image (if high risk) |
| **30 months** | **Questionnaires:** ARRQ  ***Samples/measures:*** None | None | ***Questionnaires:*** Bristol stool chart, Constipation score  ***Samples/measures:*** BP, HR, Weight, Height |
| **36 months** | Same as 12 months | Same as 12 months | ***Questionnaires:*** ADOS-T (if indicated), ASQ-TRAK, Bristol stool chart, Constipation score, Medical history, PEDI-CAT, Active play (MBQ), SACs-R, HRU, Sleep pattern (MBQ), Strippoli respiratory health questionnaire  ***Samples/measures:*** Lung function test, BP, HR, Weight, Height, FASD image (if high risk) |
| **48 months** | Same as 12 months | Same as 12 months | ***Questionnaires:*** ASQ-TRAK, PEDI-CAT, SACs-R, Medical history, Active play (MBQ), HRU  ***Measures***: BP, HR, Weight, Height, FASD image (if high risk) |
| **60 months** | Same as 12 months | Same as 12 months | Same as 48 months |

# **
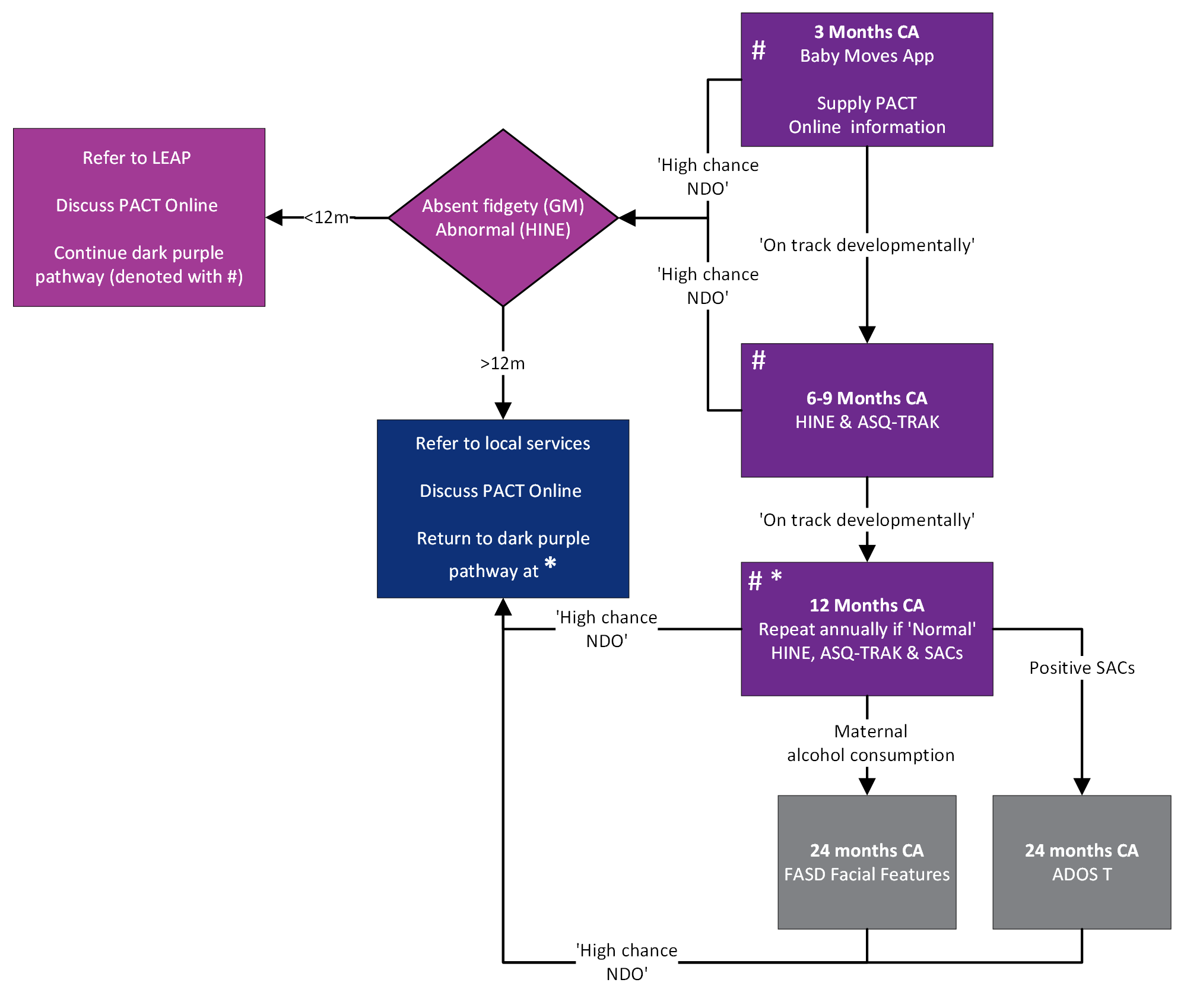
Supplementary Figure 1:** Flow chart for Infant testing and referrals in SFS.

*Abbreviations: ADOS; Autism Diagnostic Observation Schedule- Toddler, ASQ TRAK; Ages and Stages Questionnaire-Talking about Raising Aboriginal Kids, CA; corrected age; FASD; Fetal Alcohol Spectrum Disorder, GM; general movement, HINE; Hammersmith Infant Neurological Examination, LEAP; Learning through Everyday Activities with Parents, NDO; neurodevelopmental outcome, PACT Online; Parenting Acceptance and Commitment Therapy, SACs; Social Attention and Communication Surveillance*

## **References for supplementary file:**

1. Andrews G, Slade T. Interpreting scores on the Kessler Psychological Distress Scale (K10). Aust N Z J Public Health. 2001;25(6):494-7.

2. Kotz J, Munns A, Marriott R, Marley JV. Perinatal depression and screening among Aboriginal Australians in the Kimberley. Contemp Nurse. 2016;52(1):42-58.

3. Cox JL, Holden JM, Sagovsky R. Detection of postnatal depression: development of the 10-item Edinburgh Postnatal Depression Scale. The British journal of psychiatry. 1987;150(6):782-6.

4. Bruwer B, Emsley R, Kidd M, Lochner C, Seedat S. Psychometric properties of the Multidimensional Scale of Perceived Social Support in youth. Compr Psychiatry. 2008;49(2):195-201.

5. Gee G, Dudgeon P, Schultz C, Hart A, Kelly K. Aboriginal and Torres Strait Islander Social and Emotional Wellbeing. In: Dudgeon P, Milroy H, Walker R, editors. Working together : Aboriginal and Torres Strait Islander mental health and wellbeing principles and practice (2nd edition). Australia: Department of the Prime Minister Cabinet, Telethon Institute for Child Health Research, & Kulunga Research Network; 2014.

6. Gee G, Hulbert C, Kennedy H, Dwyer J, Egan J, Holmes L, et al. Development of an Aboriginal Resilience and Recovery Questionnaire – a collaboration between practitioners and help-seeking clients of a Victorian Aboriginal community controlled health service. BMC Medical Research Methodology. 2023;23(1):290.

7. Gee G, Hulbert C, Kennedy H, Paradies Y. Cultural determinants and resilience and recovery factors associated with trauma among Aboriginal help-seeking clients from an Aboriginal community-controlled counselling service. BMC Psychiatry. 2023;23(1):155.

8. He C, Salonen H, Ling X, Crilley L, Jayasundara N, Cheung HC, et al. The impact of flood and post-flood cleaning on airborne microbiological and particle contamination in residential houses. Environ Int. 2014;69:9-17.

9. Centers for Medicare and Medicaid Services. The Accountable Health Communities Health-Related Social Needs Screening Tool n.d. [cited 2025 6 January]. Available from: https://www.cms.gov/priorities/innovation/files/worksheets/ahcm-screeningtool.pdf.

10. Coates J, Anne S, Paula B. Household Food Insecurity Access Scale (HFIAS) for Measurement of Household Food Access: Indicator Guide (v. 3) Washington, D.C2007 [Available from: https://www.fantaproject.org/sites/default/files/resources/HFIAS_ENG_v3_Aug07.pdf.

11. Young SL, Boateng GO, Jamaluddine Z, Miller JD, Frongillo EA, Neilands TB, et al. The Household Water InSecurity Experiences (HWISE) Scale: development and validation of a household water insecurity measure for low-income and middle-income countries. BMJ Glob Health. 2019;4(5):e001750-e.

12. Adhikari P, Cooper-Stanbury M. Australian National Infant Feeding Survey, 2010. V1 ed: ADA Dataverse; 2018.

13. Spittle AJ, Olsen J, Kwong A, Doyle LW, Marschik PB, Einspieler C, et al. The Baby Moves prospective cohort study protocol: using a smartphone application with the General Movements Assessment to predict neurodevelopmental outcomes at age 2 years for extremely preterm or extremely low birthweight infants. BMJ Open. 2016;6(10):e013446-e.

14. Einspieler C, Prechtl HFR. Prechtl's assessment of general movements: A diagnostic tool for the functional assessment of the young nervous system. Mental Retardation and Developmental Disabilities Research Reviews. 2005;11(1):61-7.

15. Luke CR, Benfer K, Mick-Ramsamy L, Ware RS, Reid N, Bos AF, et al. Early detection of Australian Aboriginal and Torres Strait Islander infants at high risk of adverse neurodevelopmental outcomes at 12 months corrected age: LEAP-CP prospective cohort study protocol. BMJ Open. 2022;12(1):e053646.

16. Einspieler C, Bos AF, Krieber-Tomantschger M, Alvarado E, Barbosa VM, Bertoncelli N, et al. Cerebral Palsy: Early Markers of Clinical Phenotype and Functional Outcome. J Clin Med. 2019;8(10).

17. D'Aprano A, Hunter SA, Fry R, Savaglio M, Carmody S, Boffa J, et al. 'All Aboriginal and Torres Strait Islander children should have access to the ASQ-TRAK': Shared vision of an implementation support model for the ASQ-TRAK developmental screener. Health promotion journal of Australia. 2024;35(2):433-43.

18. D’Aprano A, Silburn S, Johnston V, Robinson G, Oberklaid F, Squires J. Adaptation of the Ages and Stages Questionnaire for Remote Aboriginal Australia. Qualitative Health Research. 2014;26(5):613-25.

19. Vohr BR, Wright LL, Dusick AM, Mele L, Verter J, Steichen JJ, et al. Neurodevelopmental and Functional Outcomes of Extremely Low Birth Weight Infants in the National Institute of Child Health and Human Development Neonatal Research Network, 1993-1994. Pediatrics. 2000;105(6):1216-26.

20. Haataja L, Mercuri E, Regev R, Cowan F, Rutherford M, Dubowitz V, et al. Optimality score for the neurologic examination of the infant at 12 and 18 months of age. The Journal of Pediatrics. 1999;135(2):153-61.

21. Haley S, Coster W, Dumas H, Fragala-Pinkham M, Moed R. PEDI-CAT: development, standardization and administration manual 2012 [Available from: http://www.pedicat.com/.

22. Haley SM, Coster WI, Kao Y-C, Dumas HM, Fragala-Pinkham MA, Kramer JM, et al. Lessons from Use of the Pediatric Evaluation of Disability Inventory: Where Do We Go from Here? Pediatr Phys Ther. 2010;22(1):69-75.

23. Shore BJ, Allar BG, Miller PE, Matheney TH, Snyder BD, Fragala-Pinkham M. Measuring the Reliability and Construct Validity of the Pediatric Evaluation of Disability Inventory–Computer Adaptive Test (PEDI-CAT) in Children With Cerebral Palsy. Arch Phys Med Rehabil. 2019;100(1):45-51.

24. Luyster R, Gotham K, Guthrie W, Coffing M, Petrak R, Pierce K, et al. The Autism Diagnostic Observation Schedule—Toddler Module: A New Module of a Standardized Diagnostic Measure for Autism Spectrum Disorders. J Autism Dev Disord. 2009;39(9):1305-20.

25. Astley SJ, Clarren SK. Measuring the facial phenotype of individuals with prenatal alcohol exposure: correlations with brain dysfunction. Alcohol and alcoholism (Oxford). 2001;36(2):147-59.

26. Astley SJ. Validation of the fetal alcohol spectrum disorder (FASD) 4-Digit Diagnostic Code. J Popul Ther Clin Pharmacol. 2013;20(3):e416-67.

27. Bower C, Elliott EJ. Australian Guide to the diagnosis of FASD 2020 [cited 2024. Available from: https://fasdhub.org.au/fasd-information/australian-guide-to-diagnosis-of-fasd/.

28. Trost SG, Terranova CO, Brookes DSK, Chai LK, Byrne RA. Reliability and validity of rapid assessment tools for measuring 24-hour movement behaviours in children aged 0–5 years: the Movement Behaviour Questionnaire Baby (MBQ-B) and child (MBQ-C). International Journal of Behavioral Nutrition and Physical Activity. 2024;21(1):43.

29. Strippoli M-PF, Silverman M, Michel G, Kuehni CE. A parent-completed respiratory questionnaire for 1-year-old children: repeatability. Archives of Disease in Childhood. 2007;92(10):861.

30. Barbaro J, Dissanayake C. Early markers of autism spectrum disorders in infants and toddlers prospectively identified in the Social Attention and Communication Study. Autism. 2012;17(1):64-86.

31. Barbaro J, Sadka N, Gilbert M, Beattie E, Li X, Ridgway L, et al. Diagnostic Accuracy of the Social Attention and Communication Surveillance-Revised With Preschool Tool for Early Autism Detection in Very Young Children. JAMA Netw Open. 2022;5(3):e2146415.

32. Bradley KA, DeBenedetti AF, Volk RJ, Williams EC, Frank D, Kivlahan DR. AUDIT-C as a Brief Screen for Alcohol Misuse in Primary Care. Alcohol Clin Exp Res. 2007;31(7):1208-17.

33. Dawson DA, Grant BF, Stinson FS, Zhou Y. Effectiveness of the Derived Alcohol Use Disorders Identification Test (AUDIT-C) in Screening for Alcohol Use Disorders and Risk Drinking in the US General Population. Alcoholism, clinical and experimental research. 2005;29(5):844-54.

34. Juniper EF, O'Byrne PM, Guyatt GH, Ferrie PJ, King DR. Development and validation of a questionnaire to measure asthma control. Eur Respir J. 1999;14(4):902-7.

35. Australian Government Department of Health and Ageing. The Australian type 2 diabetes risk assessment tool (AUSDRISK): Australian Government Department of Health and Ageing; 2022 [Available from: https://www.health.gov.au/resources/apps-and-tools/the-australian-type-2-diabetes-risk-assessment-tool-ausdrisk.

36. Chen L, Magliano DJ, Balkau B, Colagiuri S, Zimmet PZ, Tonkin AM, et al. AUSDRISK: an Australian Type 2 Diabetes Risk Assessment Tool based on demographic, lifestyle and simple anthropometric measures. Medical Journal of Australia. 2010;192(5):274-.

37. O'Donnell LJ, Virjee J, Heaton KW. Detection of pseudodiarrhoea by simple clinical assessment of intestinal transit rate. BMJ. 1990;300(6722):439-40.

38. Agachan F, Chen T, Pfeifer J, Reissman P, Wexner SD. A constipation scoring system to simplify evaluation and management of constipated patients. Dis Colon Rectum. 1996;39(6):681-5.

39. Anderson RM, Fitzgerald JT, Gruppen LD, Funnell MM, Oh MS. The diabetes empowerment scale-short form (DES-SF) [8]. Diabetes Care. 2003;26(5):1641-2.

40. Anderson RM, Funnell MM, Fitzgerald JT, Marrero DG. The Diabetes Empowerment Scale: a measure of psychosocial self-efficacy. Diabetes Care. 2000;23(6):739-43.

41. Heatherton TF, Kozlowski LT, Frecker RC, Fagerstrom K-O. The Fagerström Test for Nicotine Dependence: a revision of the Fagerstrom Tolerance Questionnaire. Br J Addict. 1991;86(9):1119-27.

42. Rahman AU, Mohamed MHN, Jamshed S, Mahmood S, Iftikhar Baig MA. The Development and Assessment of Modified Fagerstrom Test for Nicotine Dependence Scale among Malaysian Single Electronic Cigarette Users. J Pharm Bioallied Sci. 2020;12(Suppl 2):S671-s5.

43. Brown WJ, Burton NW, Marshall AL, Miller YD. Reliability and validity of a modified self-administered version of the Active Australia physical activity survey in a sample of mid-age women. Aust N Z J Public Health. 2008;32(6):535-41.

44. Brown WJ, Pavey T. Physical Activity in Mid-Age and Older Women: Lessons from the Australian Longitudinal Study on Women’s Health. Kinesiology review (Champaign, Ill). 2016;5(1):87-97.

45. Australian Government Department of Health Aged Care. An active way to better health: National physical activity guidelines for adults. Australian Government Publishing Service Canberra; 1999.

46. Craig CL, Marshall AL, Sjostrom M, Bauman AE, Booth ML, Ainsworth BE, et al. International Physical Activity Questionnaire: 12-Country Reliability and Validity. Med Sci Sports Exerc. 2003;35(8):1381-95.

47. Patterson R, McNamara E, Tainio M, de Sá TH, Smith AD, Sharp SJ, et al. Sedentary behaviour and risk of all-cause, cardiovascular and cancer mortality, and incident type 2 diabetes: a systematic review and dose response meta-analysis. European Journal of Epidemiology. 2018;33(9):811-29.

48. Tonkin E, Chan E, Deen C, Fredericks B, Dhurrkay M, Dissayanake HU, et al. The relative validity of the updated Menzies Remote Short-Item Dietary Assessment Tool (MRSDAT) for use with remote Aboriginal and Torres Strait Islander children and adults. BMC Public Health. 2025;25(1):1990.

49. Tonkin E, Chatfield MD, Brimblecombe J, Kleve S, Chan E, Deen C, et al. Diet quality, food security and traditional food intake of pregnant and breastfeeding women, and children 6 months to 5 years, living in eight remote Australian Aboriginal and Torres Strait Islander communities. BMC Public Health. 2025;25(1):1604.

50. Cella D, Choi SW, Condon DM, Schalet B, Hays RD, Rothrock NE, et al. PROMIS® Adult Health Profiles: Efficient Short-Form Measures of Seven Health Domains. Value in Health. 2019;22(5):537-44.

51. Monk TH, Buysse DJ, Kennedy KS, Pods JM, DeGrazia JM, Miewald JM. Measuring sleep habits without using a diary: the sleep timing questionnaire. Sleep (New York, NY). 2003;26(2):208-12.

52. Morin CM, Belleville G, Bélanger L, Ivers H. The Insomnia Severity Index: Psychometric Indicators to Detect Insomnia Cases and Evaluate Treatment Response. Sleep. 2011;34(5):601-8.

53. Roenneberg T, Wirz-Justice A, Merrow M. Life between Clocks: Daily Temporal Patterns of Human Chronotypes. Journal of biological rhythms. 2003;18(1):80-90.

54. Hopkins C, Gillett S, Slack R, Lund VJ, Browne JP. Psychometric validity of the 22-item Sinonasal Outcome Test. Clin Otolaryngol. 2009;34(5):447-54.

55. Bureau of Health Information. Aboriginal Patient Experience Question Set Sydney, NSW: BHI; 2020 [Available from: https://www.bhi.nsw.gov.au/__data/assets/pdf_file/0004/886702/BHI_Aboriginal_Patient_Experience_Question_Set.pdf.

56. Bureau of Health Information. Development Report - Aboriginal Patient Experience Question Set. Sydney (NSW): Bureau of Health Information; 2020.

57. Klima CS, Vonderheid SC, Norr KF, Park CG. Development of the Pregnancy-related Empowerment Scale. Nursing and Health. 2015.

58. Australian Curriculum Assessment and Reporting Authority (ACARA). NAPLAN 2025 Information for parents and carers 2025 [Available from: https://nap.edu.au/resources.

59. Department of Education. AEDC Data Collection Technical Report 2022 [Available from: https://www.aedc.gov.au/resources/detail/2021-aedc-data-collection-technical-report.

60. Benfer K, Boyd RN, Roe Y, Fagan R, Luke C, Mick-Ramsamy L, et al. Study protocol: peer delivered early intervention (Learning through Everyday Activities with Parents for Infants at risk of Cerebral Palsy: LEAP-CP) for First Nation Australian infants at high risk of cerebral palsy – an RCT study. BMJ Open. 2023;13(3):e059531.

61. Whittingham K, Sheffield J, Mak C, Dickinson C, Boyd RN. Early Parenting Acceptance and Commitment Therapy ‘Early PACT’ for parents of infants with cerebral palsy: a study protocol of a randomised controlled trial. BMJ Open. 2020;10(10):e037033-e.
