# Supplementary material for: Strong Families Study: protocol for a co-designed birth cohort study with Aboriginal and Torres Strait Islander families in Queensland, Australia
Source: BMJ Open. 2026 May 28;16(5):e113766. doi: 10.1136/bmjopen-2025-113766 (PMC13223655; doi:10.1136/bmjopen-2025-113766)
Supplement: Supplementary file 2 [file bmjopen-2025-113766.reviewer_comments.pdf]

PEER REVIEW HISTORY

BMJ Open publishes all reviews undertaken for accepted manuscripts. Reviewers are asked to complete a checklist review form and are provided with free text boxes to elaborate on their assessment. These free text comments are reproduced below.

ARTICLE DETAILS

Title (Provisional)

The Strong Families Study: Protocol for a Co-designed Birth Cohort Study with Aboriginal and Torres Strait Islander Families in Queensland, Australia.

Authors

Ahmed, Salma Mohamed; Dorey, Emily S; Smith, Davina; Weatherall, Loretta; Friday, Rhiannon; Massi, Luciana F; Rooney, Rebecca; Hermith-Ramirez, Diana; Kendall, Emma; Wheeler, Kai; Eades, Anne-Marie; Toombs, Maree; Boyd, Roslyn N; Marriott, Rhonda; Eades, Sandra J; Benfer, Katherine; Reid, Natasha; Whittingham, Koa; Ware, Robert S.; Martin, Elizabeth; Robinson, Paul D; Clifton, Vicki; Callaway, Leonie Kaye; Kumar, Sailesh; Strong Families, Indigenous Steering Committee; Rae, Kim

---

VERSION 1 - REVIEW

**Reviewer**	**1**
**Name**	**Zengin, Ayse**
**Affiliation**	**Monash University, School of Clinical Sciences**
**Date**	**10-Feb-2026**
**COI**	**None**

This manuscript presents the protocol for a large, Indigenous-led, co-designed prospective birth cohort study following Aboriginal and Torres Strait Islander families from pregnancy to early childhood, with embedded family support programs. The study addresses a critical gap in Australian perinatal and early-life research by embedding Indigenous governance, Indigenous data sovereignty, and strengths-based methodologies.

Overall, this is a high-quality, well-justified, and ethically robust protocol that aligns strongly with BMJ Open's aims for transparency and methodological clarity in protocol publication. The Indigenous leadership, governance framework, and co-design processes represent a major strength and advance best practice in Indigenous health research. This protocol excels in Indigenous governance and ethical considerations. The clear articulation of the Indigenous Steering Committee's role, community control of data, and culturally safe procedures

represents a strength that aligns well with current best practice. This section may serve as a template for future Indigenous health research protocols.

There are a few important methodological clarifications that are required, to ensure the protocol is sufficiently detailed and reproducible. See below:

1. Clarify how co-creation with communities will continue throughout data interpretation and outputs, not only during inception and governance. For example: will data analysis plans be co-developed with community partners? How will communities be involved in determining strengths-based outcomes and dissemination products?

2. The protocol currently presents a broad set of domains but does not define clear primary and secondary outcomes. For transparency, authors should state the primary outcomes that will guide sample size and analysis. Provide specific examples of how “strengths” will be measured, analysed, and reported (e.g. validated resilience scales, cultural connectedness indices), including plans to ensure these measures reflect community values. Also, authors should clarify how culturally adapted instruments were selected and validated, and whether community feedback informed scoring or interpretation.

3. A formal rationale for the target sample size (n=400 families) is needed. Although, BMJ Open protocols do not need to provide power calculations for every outcome, justifying how this sample size will support key analyses (especially for primary outcomes, longitudinal modelling, and nested program evaluation), improve transparency and will strengthen the paper. It will also help if the authors could provide details on the analysis plan including, handling repeated measures and clustering by site/community, and potential strategies for handling attrition and missing data (e.g. multiple imputation, weighting). Given anticipated movement and follow-up challenges, retention strategies should be described.

4. Given the intensity and duration of follow-up (pregnancy to 5 years), attrition is a major risk. While acknowledged as a limitation, the protocol does not specify the expected attrition rates and potential retention strategies beyond incentives - these would strengthen the manuscript.

5. The study includes two family support programs (PACT Online, LEAP). The manuscript should clearly define the analysis plan for these as to how will families participating in these programs be compared with those who do not? And what adjustments will be made for potential selection bias (e.g. families referred may differ systematically)? Also, specify how RE-AIM indicators will be measured and reported.

6. A flow diagram illustrating participant engagement from pregnancy through early childhood—including data collection points and nested program participation—would aid readability. Sup Table 1 is extremely useful, but a more concise flow diagram in the manuscript would be beneficial.

7. Sup Fig 2: NDO is not defined in caption

8. Is there a reason as to why the study was not registered (e.g. ANZCTR)? or have the details simply not been inserted into the manuscript?

VERSION 1 - AUTHOR RESPONSE

**Reviewer: 1**<br>**Dr. Ayse Zengin, Monash University**<br>**Comments to the Author:**	**Comments/Suggestions addressed**
This manuscript presents the protocol for a large, Indigenous-led, co-designed prospective birth cohort study following Aboriginal and Torres Strait Islander families from pregnancy to early childhood, with embedded family support programs. The study addresses a critical gap in Australian perinatal and early-life research by embedding Indigenous governance, Indigenous data sovereignty, and strengths-based methodologies.<br><br>Overall, this is a high-quality, well-justified, and ethically robust protocol that aligns strongly with BMJ Open's aims for transparency and methodological clarity in protocol publication. The Indigenous leadership, governance framework, and co-design processes represent a major strength and advance best practice in Indigenous health research. This protocol excels in Indigenous governance and ethical considerations. The clear articulation of the Indigenous Steering Committee's role, community control of data, and culturally safe procedures represents a strength that aligns well with current best practice. This section may serve as a template for future Indigenous health research protocols.	Thank you sincerely for your thoughtful and encouraging comments. We deeply appreciate the care you took in assessing our manuscript and providing your feedback. Your acknowledgement of the work undertaken by our team, together with the Indigenous Steering Committee and community is genuinely valued. Your reflections have been motivating for the team and reinforce our commitment to conducting research that is both rigorous, culturally safe and aligned with priorities of Aboriginal and Torres Strait Islander community. Thank you again for your time and insight in strengthening this manuscript.<br><br>All specific comments are addressed below.
There are a few important methodological clarifications that are required, to ensure the protocol is sufficiently detailed and reproducible. See below:<br><br>1. Clarify how co-creation with communities will continue throughout data interpretation and outputs, not only during inception and governance. For example: will data analysis plans be co-developed with community partners? How will communities be involved in determining strengths-based outcomes and dissemination products?	Community co-creation/co-design continues throughout data interpretation and outputs in several ways. First, on behalf of their communities, the Indigenous Steering Committee (ISC) has prioritised the health themes and culturally appropriate measures that will guide what analyses will be undertaken. These health themes and measures have been illustrated in Fig. 3 of the manuscript. Also, any proposed sub-study involving new analyses must be reviewed and endorsed by the ISC before progressing to the Chief Executive Committee, ensuring the ISC maintains authority over which research

	questions, datasets and analytical approaches are permitted (Line 245 – 248, page 11). Through this process, the ISC confirms that analyses focus on strengths-based outcomes aligned with community priorities and cultural values. The ISC will also review all manuscripts, reports and dissemination products prior to release to ensure interpretations are culturally safe, contextually grounded and free from deficit-based framing. Furthermore, SFS Indigenous Research Team members are an integral part of the study's implementation evaluation, including co-leading all aspects of instrument design, data collection, analysis and writing.
2. The protocol currently presents a broad set of domains but does not define clear primary and secondary outcomes. For transparency, authors should state the primary outcomes that will guide sample size and analysis. Provide specific examples of how "strengths" will be measured, analysed, and reported (e.g. validated resilience scales, cultural connectedness indices), including plans to ensure these measures reflect community values. Also, authors should clarify how culturally adapted instruments were selected and validated, and whether community feedback informed scoring or interpretation.	The primary outcome refers to the "chance of cerebral palsy/ autism spectrum disorder/ neurodevelopmental outcome" in children over the study period (Line numbers 400 – 401, pages 16). Rather than defining secondary outcomes, themes such as maternal wellbeing, family strengths, and social, environmental and health service factors are considered exposures or background factors, which reflects the exploratory nature of a longitudinal study.<br><br>All measurement tools were selected and endorsed by the ISC based on cultural relevance, acceptability and alignment with community values. These include validated, culturally adapted instruments and culturally specific tools that have been described in-depth in the supplementary material. Each instrument has established scoring and interpretation guidelines, which the study will follow. Strengths will be analysed through the domains within these measures. Analyses will combine descriptive summaries with modelling approaches to explore how strengths relate to maternal, paternal, infant and child health outcomes, consistent with a strengths-based and non-deficit approach.<br><br>Cultural adaptation and validation processes were informed by ISC review and, where applicable, previous Indigenous-led research. Community and ISC feedback guided the selection of instruments, refinement of wording, and assessment of cultural fit. Ongoing ISC involvement during analysis and reporting will ensure interpretation reflects community values and lived experience. This will include guidance on language of results, the

	cultural meaning of scores, and appropriate framing of findings. A separate publication is underway that elaborates the co-design process and how the ISC selected the health themes and measurement tools to be used in the study.
3. A formal rationale for the target sample size (n=400 families) is needed. Although, BMJ Open protocols do not need to provide power calculations for every outcome, justifying how this sample size will support key analyses (especially for primary outcomes, longitudinal modelling, and nested program evaluation), improve transparency and will strengthen the paper. It will also help if the authors could provide details on the analysis plan including, handling repeated measures and clustering by site/community, and potential strategies for handling attrition and missing data (e.g. multiple imputation, weighting). Given anticipated movement and follow-up challenges, retention strategies should be described.	Thank you. We have strengthened the Methods and Analysis section to provide a clearer rationale for n=400 and to expand the statistical plan. Specifically, we added a dedicated Sample size and statistical power subsection (assumptions for the primary outcome, detectable effect sizes for binary and continuous outcomes, and the conservative nature of single time-point assumptions in a repeated-measures design). We also expanded the Statistical analysis section to specify: (i) use of mixed-effects models to handle repeated measures (with time modelled where applicable), (ii) accounting for clustering by site/community given the multi-site teletrial design (e.g. site/community random effects and/or robust standard errors, depending on outcome/model), and (iii) strategies for attrition and missing data, including multiple imputation where plausible and sensitivity analyses (including weighting-based approaches if differential loss to follow-up is observed). Finally, we added a brief Retention strategies paragraph describing flexible follow-up approaches (in-person preferred with a phone option), reminders, and site-approved participant appreciation/incentives to support ongoing engagement over time.<br><br>Line numbers 396 – 423, pages 16 and 17:<br><br>Sample size and statistical power<br>The sample size of 400 families followed prospectively was chosen to provide sufficient power to examine associations between perinatal strengths/risk factors and pregnancy, birth and early childhood outcomes. Assuming approximately 5% of the cohort will have the primary outcome of “chance of cerebral palsy/ autism spectrum disorder/ neurodevelopmental outcome”, this sample gives 80% power (α=0.05) to detect absolute differences of ≥8 percentage points between binary risk factor groups. For more common binary outcomes, differences of >28% can be detected. For continuous outcomes, we will be able to identify between-category differences of >0.3 SD (Z-score units). These estimates are

	conservative, as repeated measurements over time and mixed effects models will further improve precision.<br><br>Statistical analysis<br>To examine relationships between perinatal strengths/risk factors and continuous outcomes (e.g. KMMS), we will use linear or mixed effects regression for repeated measures. Binary outcomes will be analysed using logistic regression, and count outcomes with Poisson or other suitable count models. Analyses will account for the multi-site teletrial design by adjusting for clustering at the site/community level (e.g. through random or fixed effects, or robust standard errors). For multivariable modelling, candidate predictors will be screened individually, with final model selection guided by Bayesian Information Criteria. Model calibration will be checked graphically, and internal validation performed via bootstrap resampling.<br><br>Missing data will be treated case-by-case using multiple imputation when data are plausibly missing at random, and pattern mixture methods with sensitivity analyses when not missing at random mechanisms are likely. Longitudinal mixed effects models will use all available observations under missing at random assumptions. If differential loss to follow up occurs, we will apply weighting based sensitivity analyses (e.g., inverse probability of follow up weighting). Each analysis will include appropriate adjustments for multiple comparisons to balance type I and II error risks.<br><br>We have also included more information regarding retention strategies: Line numbers 385 - 395, page 16<br><br>To support retention over the five-year follow-up, we will prioritise in-person, culturally safe data collection using a yarning approach, supported by flexible scheduling, telehealth or phone options, and alignment with existing antenatal or paediatric appointments. Retention will be strengthened through sustained, trusting relationships fostered by ACCHOs and partnering hospitals, regular personalised contact (SMS, calls, emails, periodic check-ins), and routine updates to contact details, including alternative contacts. Reminder messages will be sent for upcoming visits, and site-approved appreciation or

	incentives will be offered where appropriate. We will also create a positive participant experience by ensuring visits are welcoming and culturally respectful, and by acknowledging family and child milestones to support ongoing engagement.
4. Given the intensity and duration of follow-up (pregnancy to 5 years), attrition is a major risk. While acknowledged as a limitation, the protocol does not specify the expected attrition rates and potential retention strategies beyond incentives - these would strengthen the manuscript.	We agree that attrition is a key risk in a long-term cohort study. Based on the small number of longitudinal cohorts involving Aboriginal and Torres Strait Islander families, we anticipate an attrition rate in the range typically observed in similar studies, with the possibility of gradual increases over time. To mitigate this risk, we have strengthened our description of retention strategies beyond incentives, as mentioned in the previous response in Line numbers 385 - 395, page 16. These include building strong, trusting relationships with families through sustained engagement by Aboriginal Community Controlled Health Organisations (ACCHOs) and partnering hospitals, maintaining regular and meaningful contact through SMS, calls, email reminders and periodic check-ins even between scheduled visits, and offering flexibility through telehealth options and aligning study visits with existing health appointments (e.g., antenatal or paediatric clinic visits). We also aim to create a positive and culturally safe participant experience, celebrating family (e.g. family/baby portraits) and child milestones and ensuring that study visits feel supportive and welcoming. These combined strategies will help support continued engagement throughout the five-year follow-up period.
5. The study includes two family support programs (PACT Online, LEAP). The manuscript should clearly define the analysis plan for these as to how will families participating in these programs be compared with those who do not? And what adjustments will be made for potential selection bias (e.g. families referred may differ systematically)? Also, specify how RE-AIM indicators will be measured and reported.	We added a paragraph describing the evaluation plan for PACT Online and LEAP. The effectiveness of the PACT Online and LEAP programs will be assessed using longitudinal mixed-effects models with covariate adjustment. In both cases participation in the programs will be the main effect. Differences in characteristics of families who do, and do not, participate in the programs will be assessed. If any differences exist they will be adjusted for in analyses using weighting methods such as propensity matching.<br><br>Line numbers 424 – 430, page 17:<br><br>For the PACT Online and LEAP programs, effectiveness will be investigated using mixed effects models with program participation included as the main effect and models adjusted

	for baseline factors. Differences in characteristics of families who do, and do not, participate in the programs will be assessed. If any differences exist, they will be adjusted for in analyses using weighting methods such as propensity matching. Cohort embedded summaries of eligibility, referral and uptake will be reported descriptively. The sensitivity of observational comparisons (e.g., uptake vs non uptake) will be assessed.<br><br>Regarding RE-AIM, we have expanded on the measures and reporting. Line 431 – 441, page 17:<br><br>An implementation and evaluation analysis will be undertaken and reviewed by the ISC. The RE-AIM framework will be applied to assess the reach, effectiveness, adoption, implementation and maintenance of SFS across all the study sites. A combination of quantitative metrics (e.g., engagement numbers, training outcomes) and qualitative insights from interviews and focus groups will be utilised. The evaluation will draw on study monitoring data, staff training assessments, participant surveys and yarning interviews to measure how effectively the study is implemented, delivered and taken up. This process ensures comprehensive staff training, strategic resource allocation and identification of site-specific needs, while maintaining consistent adherence to the study protocol. Findings for each RE-AIM domain will be summarised using descriptive quantitative indicators and thematic analysis of qualitative data.
6. A flow diagram illustrating participant engagement from pregnancy through early childhood—including data collection points and nested program participation—would aid readability. Sup Table 1 is extremely useful, but a more concise flow diagram in the manuscript would be beneficial.	Thank you for the suggestion. We have now included an additional figure to illustrate the study visits which will complement Sup Table 1.
7. Sup Fig 2: NDO is not defined in caption	This has now been revised to refer to ‘Neurodevelopmental Outcomes’
8. Is there a reason as to why the study was not registered (e.g. ANZCTR)? or have the details simply not been inserted into the manuscript?	Strong Families Study (SFS) has not been registered as it is a prospective observational birth cohort and not a clinical trial. However, LEAP (registration number: ACTRN12619000969167) and PACT Online (ACTRN12618000986279) which are the nested family support programs within SFS are already registered.

VERSION 2 - REVIEW

Reviewer 1

Name Zengin, Ayse

Affiliation Monash University, School of Clinical Sciences

Date 23-Apr-2026

COI

Authors have addressed all comments to a standard that has improved the paper immensely - great work!
